# Longitudinal Natural History Study of Children and Adults with Rare Solid Tumors: Initial Results for First 200 Participants

**DOI:** 10.1158/2767-9764.CRC-23-0247

**Published:** 2023-12-06

**Authors:** Shadin Ahmed, Mary Frances Wedekind, Jaydira Del Rivero, Margarita Raygada, Robin Lockridge, John W. Glod, Crystal Flowers, BJ Thomas, Donna B. Bernstein, Oxana B. Kapustina, Ashish Jain, Markku Miettinen, Mark Raffeld, Liqiang Xi, Manoj Tyagi, Jung Kim, Kenneth Aldape, Ashkan A. Malayeri, Rosandra N. Kaplan, Taryn Allen, Christina A. Vivelo, Abby B. Sandler, Brigitte C. Widemann, Karlyne M. Reilly, for the MyPART network

**Affiliations:** 1 Pediatric Oncology Branch, Center for Cancer Research, NCI, Bethesda, Maryland.; 2 Developmental Therapeutics Branch, Center for Cancer Research, NCI, Bethesda, Maryland.; 3 Clinical Research Directorate (CRD), Frederick National Laboratory for Cancer Research, Frederick, Maryland.; 4 Research Computing, Department of Information Technology, Boston Children's Hospital, Boston, Massachusetts.; 5 Laboratory of Pathology, Center for Cancer Research, NCI, Bethesda, Maryland.; 6 Department of Radiology and Imaging Sciences, Clinical Center, NIH, Bethesda, Maryland.; 7 Kelly Government Solutions, Bethesda, Maryland.

## Abstract

Understanding of tumor biology and identification of effective therapies is lacking for many rare tumors. My Pediatric and Adult Rare Tumor (MyPART) network was established to engage patients, advocates, and researchers and conduct a comprehensive longitudinal Natural History Study of Rare Solid Tumors. Through remote or in-person enrollment at the NIH Clinical Center, participants with rare solid tumors ≥4 weeks old complete standardized medical and family history forms, patient reported outcomes, and provide tumor, blood and/or saliva samples. Medical records are extracted for clinical status and treatment history, and tumors undergo genomic analysis. A total of 200 participants (65% female, 35% male, median age at diagnosis 43 years, range = 2–77) enrolled from 46 U.S. states and nine other countries (46% remote, 55% in-person). Frequent diagnoses were neuroendocrine neoplasms (NEN), adrenocortical carcinomas (ACC), medullary thyroid carcinomas (MTC), succinate dehydrogenase (SDH)-deficient gastrointestinal stromal tumors (sdGIST), and chordomas. At enrollment, median years since diagnosis was 3.5 (range = 0–36.6), 63% participants had metastatic disease and 20% had no evidence of disease. Pathogenic germline and tumor mutations included *SDHA/B/C* (sdGIST), *RET* (MTC), *TP53* and *CTNNB1* (ACC), *MEN1* (NEN), and *SMARCB1* (poorly-differentiated chordoma). Clinically significant anxiety was observed in 20%–35% of adults. Enrollment of participants and comprehensive data collection were feasible. Remote enrollment was critical during the COVID-19 pandemic. Over 30 patients were enrolled with ACC, NEN, and sdGIST, allowing for clinical/genomic analyses across tumors. Longitudinal follow-up and expansion of cohorts are ongoing to advance understanding of disease course and establish external controls for interventional trials.

**Significance:**

This study demonstrates that comprehensive, tumor-agnostic data and biospecimen collection is feasible to characterize different rare tumors, and speed progress in research. The findings will be foundational to developing external controls groups for single-arm interventional trials, where randomized control trials cannot be conducted because of small patient populations.

## Introduction

Rare tumors and cancers occur in <15 incident cases per 100,000 per year. By this definition in the United States, all pediatric tumors (<20 years) are rare and <13% of tumors diagnosed in adults (≥20 years) are rare (1). Hereafter, we use the term “rare tumors” to include both rare tumors and rare cancers. The overall burden of rare tumors is significant with 27% of U.S. tumor diagnoses being rare. Rare solid tumors are understudied and knowledge of their biology, genomics, clinical course, and psychologic impact is limited. For many rare tumors, no effective therapy exists, and clinical trials are difficult to conduct. Several programs have begun to address obstacles in rare tumor research (2–9), such as designing effective interventional trials (10–13), developing new models (14) and identifying new therapeutic targets (1, 15); however, there remains a large unmet need to study rare tumors comprehensively and longitudinally. **M**y **P**ediatric and **A**dult **R**are **T**umor (MyPART) network (https://www.cancer.gov/pediatric-adult-rare-tumor/) was established at the NCI, Center for Cancer Research (CCR) in response to NCI Cancer Moonshot^SM^ recommendations to increase patient engagement in cancer research (16). MyPART is a team of clinicians and researchers partnering with advocates, patients, and their families to accelerate rare solid tumor research, focusing on patient experience over time through development of the **N**atural **H**istory and Biospecimen Acquisition Study for Children and Adults with **R**are **S**olid **T**umors (NHRST; NCT03739827; ref. 17).

Natural history studies are critical to understanding rare tumors. Draft guidance by the FDA provides specifics on leveraging natural history studies in the drug development process for patients with rare diseases, to create external control groups in instances where randomized clinical trials are difficult to conduct due to few enrollees (18). For example, regulatory approval of selumetinib for plexiform neurofibromas (PN) relied on natural history data of PN growth rates to demonstrate that selumetinib significantly altered tumor growth trajectory (19, 20). In addition, the FDA recently described how external controls contributed to regulatory approvals for several targeted therapies (21). The goal of NHRST is to develop deep understanding of the clinical course and pathogenesis of rare solid tumors of unmet need, translating findings to improve outcomes for patients with rare solid tumors. Here we report initial results from baseline evaluation of the first 200 participants.

## Materials and Methods

### Study Design and Patient Eligibility

This single-institution NHRST study was conducted in accordance with recognized ethical guidelines as per The Belmont Reports and the Department of Health and Human Services Common Rule and was approved by the NIH Institutional Review Board. Written informed consent was obtained from all participants 18 years and older or their legally authorized representative, and from parent(s) of minors. Verbal assent was obtained as appropriate from participants 7–17 years of age. Consent and assent language was approved by the NIH Institutional Review Board. Patients ≥4 weeks old with a rare solid tumor (<15 cases in 100,000 people per year), a genetic cancer predisposition syndrome (GCPS), or their family members were eligible for participation. Of note, the study focuses on enrolling participants with very rare tumors, defined as <2 per million per year, rare tumors that lack clinical trials or standard therapy, tumors that are rare in the pediatric population even if they are common in adults and unusual presentations of rare tumors (2). Participants could enroll remotely (field cohort) or in person at the NIH Clinical Center (NIHCC; clinic cohort). Clinic cohort patients underwent clinical evaluation at NIHCC. The detailed study design, data collection methods and all study forms are provided in Wedekind and colleagues (17).

### Recruitment

To increase public awareness, facilitate referrals, and provide educational information, a website was established (www.cancer.gov/mypart) providing information on rare solid tumors, advocacy partnerships, and a lay study description.

### Data Collection

NHRST collects all available medical history, including imaging studies and pathology specimens retrospectively and prospectively. Participants (or their caregivers) are asked to complete standardized forms on medical, behavioral, and family tumor history. Follow-up forms are sent to participants yearly in the month of their original consent. Clinicians review yearly medical records and extract data on pathology, presenting symptoms, and treatment by event.

### Clinical Evaluations for the Clinic Cohort

For participants coming to NIHCC, clinical phenotyping, clinical laboratory studies, and imaging are conducted, as well as germline genotyping as indicated. A senior genetic counselor (M. Raygada) constructs pedigrees using Progeny Clinical—v.10.6.2.0 (Progeny Genetics LLC. 2019).

### Patient Reported Outcomes

The Patient-Reported Outcome Measurement Information System (PROMIS; https://commonfund.nih.gov/promis/tools) is used to collect patient reported outcomes (PRO) from adult/pediatric participants and pediatric caregivers. Encrypted patient responses are converted to standard T-scores through the Assessment Center-Application Programming Interface (https://staging.healthmeasures.net/implement-healthmeasures/administration-platforms/assessment-center-api), such that mean = 50 and SD = 10. Patients are reminded 4 weeks after initial request to complete PROs and data are considered missing if participants do not complete forms within 6 weeks after initial contact. For patients unable to complete forms independently due to illness or preference, PROs may be administered by videoconference by a clinical psychologist (R. Lockridge). Anyone with a depression score >70 receives a follow-up call by a psychologist or member of the behavioral health team.

### Data Storage

All standardized forms and PROs are entered into a secure, custom Labmatrix database (Biofortis). Participant and caregiver-reported data are collected electronically using Scribe developed by NCI CCR Center for Information Technology (CIT).

### Biospecimen Collection and Analysis

Participants provide a saliva sample and clinic cohort participants provide blood samples. Tumor diagnosis is confirmed by NIHCC pathologists. Patients with sufficient pathology specimens have tumor molecular analysis performed, using either gene panel sequencing of 500+ genes [Trusight Oncology 500 (TSO500), Illumina] plus RNA sequencing (RNA-seq) exome fusion analysis (Supplementary Table S1), tumor-normal matched whole-exome sequencing (T-N WES; available starting in second year of accrual), and/or specific molecular analyses based on tumor type, for example, detection of characteristic gene fusions. The current somatic analysis is limited to TSO500 small nucleotide variants and does not include large gene amplifications/deletions (e.g., brachyury) or fusions. Somatic variants were classified from TSO500 gene panel sequencing data using QIAGEN (RRID:SCR_008539) Clinical Insights Interpret (QCI) by the NIHCC Laboratory of Pathology and compiled for the analysis presented here. Because RNA-seq exome fusion and copy-number variation (CNV) calling was not initially available, fusions and CNVs are not included in this analysis. Participants also get clinical-grade germline sequencing as clinically indicated through commercial vendors (GeneDx or Invitae) or T-N WES (Laboratory of Pathology, CCR, NCI). The current germline analysis was compiled from all available sources of germline genotypes. Germline variants for T-N WES performed at NIHCC were called using the NCI Oncogenomics platform and the ClinOmics analysis pipeline developed by Dr. Javed Khan's group (NCI, CCR). Variants scored as Path/LP by Clinvar or Intervar were selected and those with a variant allele frequency (VAF) of ≥25% were included in the analysis. Pathogenicity was classified as pathogenic (Path), likely pathogenic (LP), and variants of uncertain significance (VUS) while actionability was tiered as 1A, 1B, 2C, 2D, and 3 according to Association for Medical Pathology (AMP)/American Society of Clinical Oncology/College of American Pathologists (CAP) guidelines (22). Participants are provided recommendations about management, treatment options, and relevant available clinical trials, either at NIHCC or outside sites.

### Data Analysis

Data linked to the first 200 enrollees were downloaded from Labmatrix, the NIH (BTRIS), NCI Oncogenomics platform, and NIH Integrated Data Analysis Platform (NIDAP). Data were validated, cleaned, and combined using Excel v16 for Mac (Microsoft). Prism 9 for Mac (GraphPad, RRID:SCR_002798) was used to graph data and analyze statistics. SSPS v28 (IBM) was used to analyze PRO results. Data entered as free text were further manually curated after download.

Participant home zip codes were converted to longitude and latitude, using data from U.S. Zip Code Database (https://www.unitedstateszipcodes.org/zip-code-database/) or from web searches for international participants. Distance from the participants home to NIHCC was estimated using the formula D = SQRT[(Latitude_home_ − Latitide_NIHCC_)^2^ + (Longitude_home_ − Longitude_NIHCC_)^2^] and the distributions for clinic and field groups compared using two-tailed Mann–Whitney test.

Race and ethnicity data were collected through medical records and self-reports. Clinical data were extracted from medical records (treatments received) or recorded when patients came to clinic (performance scores, height, weight, substance use). Body mass index (BMI) [participants ≥20 years (23)] or BMI percentile (participants <20 years old; (https://www.cdc.gov/healthyweight/bmi/widget/calculator.html) were calculated at baseline. U.S. population BMI ranges were taken from the National Health and Nutrition Examination Survey (NHAHES) prepandemic data (24, 25). Occupation, education levels, and cigarette exposure were from self-reports. Drug names were standardized to generic name and classified according to the Cancer Drug Pharmacology Table from the BC Cancer Pharmacology Education Program (26) as cytotoxic chemotherapy, hormonal therapy, immunotherapy, and targeted therapy.

PRO measures of pain, depression, and anxiety were analyzed for adult subjects only. Although pediatric patients (ages 8–17) and their caregivers were also administered PROMIS measures (RRID:SCR_004718), fewer than 10 participant responses were collected out of 29 possible pediatric participants and results were not analyzed at this time. In accordance with PROMIS T-score threshold guidance, participants with T-scores greater than 1 SD above the mean were considered to have clinically significant (CS) levels of pain, depression, and/or anxiety (27, 28).

### Data Availability

Data are available through controlled access at dbGAP (RRID:SCR_002709) accession phs003143.v1.p1 (http://www.ncbi.nlm.nih.gov/projects/gap/cgi-bin/study.cgi?study_id=phs003143.v1.p1).

## Results

### Enrollment and Participant Demographics

**FIGURE 1:**
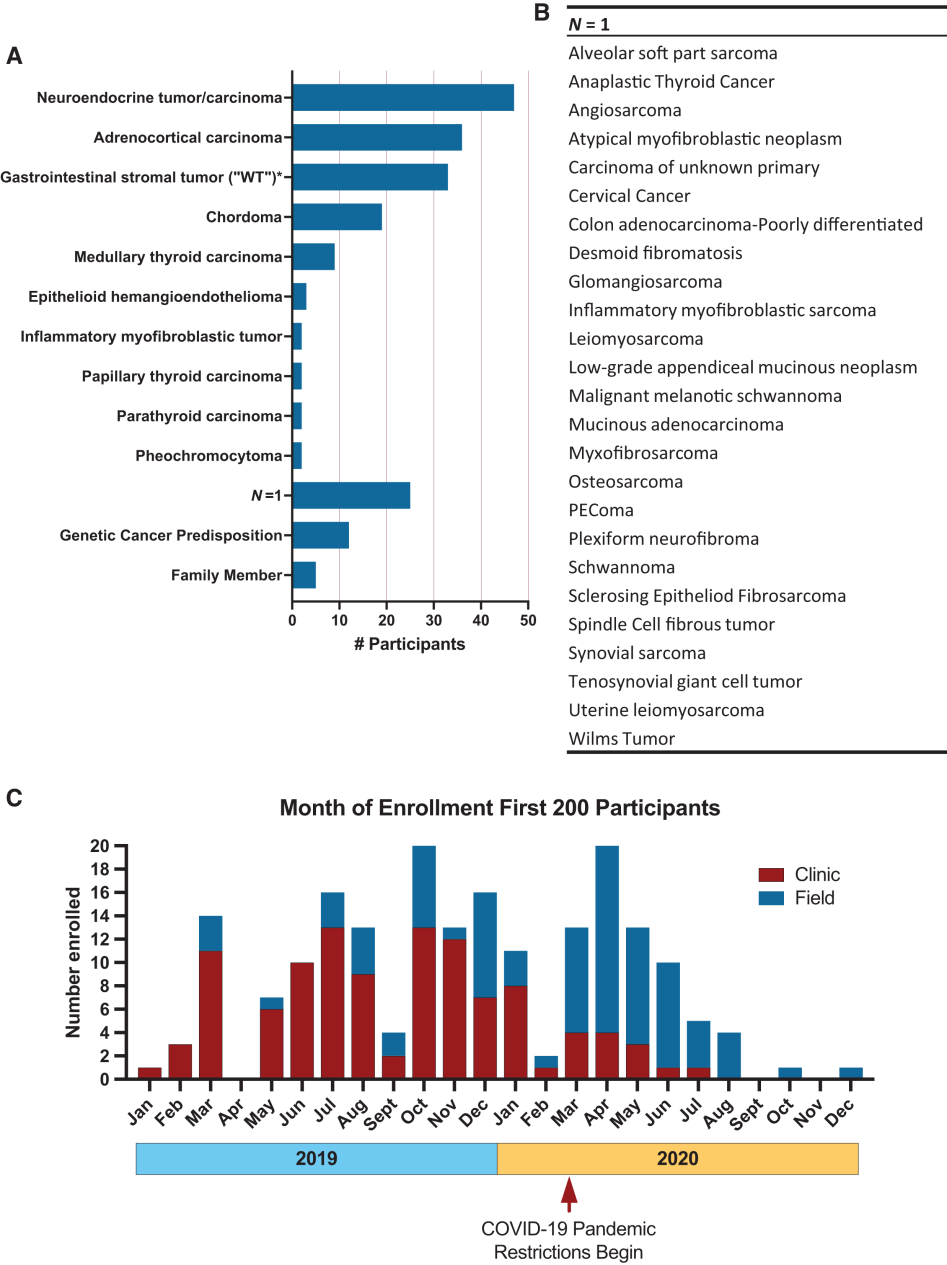
**A,** Number of participants by rare tumor diagnoses (*N* = 197). **B,** Diagnoses with only one participant enrolled (*N* = 1). **C,** Month of enrollment of 197 participants in 2019 and 2020, relative to the start of COVID pandemic restrictions at NIHCC in March 2020. Clinic cohort patients are shown in red and field cohort patients are in blue.

Between January 2019 and December 2020, we enrolled 200 NHRST participants. Three participants withdrew (all unaffected family members of one affected participant) leaving 197 participants for analysis. The most frequent tumor type was NEN (24%), followed by ACC (18%), succinate dehydrogenase (SDH)-deficient gastrointestinal stromal tumor (sdGIST; 17%), chordoma (10%), and MTC (5%; Table 1; Fig. 1A). Participants with GCPS and biological relatives of patients with rare tumors comprise 9% of enrollees. A substantial proportion of participants (13%) were the only enrollee with their tumor type in the first 200 participants (Fig. 1B).

**TABLE 1:** Demographics and clinical characteristics of first 200 enrollees showing number of participants in each category

	All	ACC	NEN	MTC	GIST	Chord	Other^a^	Mut Carr	Fam Memb
Total subjects	200	36	47	9	33	19	36	12	8
Consent withdrawn	0	0	0	0	0	0	0	0	3
Subjects with data analyzed	197	36	47	9	33	19	36	12	5
Sex									
Male	68	5	19	1	14	9	14	2	4
Female	129	31	28	8	19	10	22	10	1
Age at diagnosis (yrs)									
<18	29	2	1	1	7	11	7	N/A	N/A
≥18, <40	54	11	4	6	14	4	15	N/A	N/A
≥40	97	23	42	2	12	4	14	N/A	N/A
Race									
White	150	34	36	4	26	7	31	10	2
Black/African American	9	0	2	2	2	1	2	0	0
Asian	9	0	0	1	2	3	2	0	1
Pacific Islander^b^	1	0	0	0	1	0	0	0	0
Mixed	10	1	4	1	1	1	0	2	0
Not reported	18	1	5	1	1	7	1	0	2
Enrollment type									
Clinic	109	26	20	8	21	10	20	4	0
Field	88	10	27	1	12	9	16	8	5
Days dx to enrl Median (range)	1,311 (0–13,366)	1,349 (56–8,350)	1,391 (18–7,035)	3,057 (481–13,129)	2,029 (0–13,366)	882 (22–6,302)	376 (2–5,727)	N/A	N/A
Disease status at diagnosis									
Localized	103	24	15	4	22	15	23	N/A	N/A
Metastatic	73	12	32	5	10	3	11	N/A	N/A
Not available	4	0	0	0	1	1	2	N/A	N/A
Disease status at enrollment									
NED	36	5	8	2	7	7	7	N/A	N/A
Localized	31	4	3	0	8	6	10	N/A	N/A
Metastatic	113	27	36	7	18	6	19	N/A	N/A
Clinic cohort patients: Performance score – Median, (range, *N*)									
Karnofsky (≥16yrs)	90 (50–100, 86)	90 (70–100, 24)	90 (60–100, 20)	90 (80–100, 8)	90 (70–100, 21)	80 (70–100, 5)	95 (50–100, 20)	90 (80–100, 2)	N/A
Lansky (<16 yrs)	100 (90–100, 5)	100 (100, 2)	N/A	N/A	N/A	100 (90–100, 5)	N/A	100 (100, 2)	N/A
Cancer in first-degree relative									
Yes	93	19	21	5	11	7	13	12	5
No	99	15	25	4	22	12	21	0	0
Not reported	5	2	1	0	0	0	2	0	0

Abbreviations: ACC, adrenocortical carcinoma; Chord, chordoma; dx, diagnosis; enrl, enrollment; Fam Memb, family member; GIST, gastrointestinal stromal tumor; MTC, medullary thyroid carcinoma; Mut Carr, mutation carrier; NED, no evidence of disease; NEN, neuroendocrine neoplasm (includes neuroendocrine carcinoma); yrs, years.

^a^Other is the combined data for all other tumor types, not ACC, chordoma, GIST, MTC, and NET.

^b^Includes Native Hawaiians and Pacific Islanders; American Indian or Alaskan Natives were not identified in the cohort.

We enrolled more females (65%), reflecting female predominance in some of the tumor types enrolled, such as ACC and sdGIST (Table 1). Participants with ACC were almost entirely female (86%). We enrolled children, adolescents and young adults (AYA), and older adults for several tumor types from 46 U.S. states, and nine countries across six continents (Supplementary Fig. S1A and S1B); however, particular tumors were more frequently pediatric (e.g., chordoma) or adult (e.g., ACC; Table 1; Supplementary Fig. S2).

We found 14% discrepancy in race between participant-reported data and medical record extraction, with neither source being complete for all participants. Notably, race could be assigned to more participants when self-reported data were combined with medical record data (Supplementary Fig. S3A and S3B), and self-reported data demonstrated minority racial backgrounds within parent ancestry (Supplementary Fig. S3C). Self-reported race was preferentially used if a discrepancy was noted. Table 1 summarizes compiled race information.

Until March 2020, when COVID-19 pandemic restrictions began at NIHCC, most participants were in the clinic group, whereas during pandemic restrictions, most were in the field group due to restrictions on bringing patients to NIHCC (Fig. 1C). Remote participation thus allowed for continued enrollment during the pandemic, although at a reduced capacity, particularly during the last quarter of 2020. In addition, during the fall 2020, there was a pause in enrollment to validate and analyze data. There was no significant difference between field and clinic group in how close they lived to NIHCC (Supplementary Fig. S4A and S4B) over the time of enrollment for the first 200 enrollees. However, during the pandemic the field cohort enrolled from significantly further away (Supplementary Fig. S4C). Form completion by clinic participants was significantly greater than field participants (Supplementary Fig. S5). Overall, the ability to collect data using our forms ranged from 43% for the pain interference index in the field cohort to 98% for medical record extraction.

Most participants did not enroll at the time of diagnosis, but a median of 3.5 years (range = 0–36.3) past initial diagnosis (Supplementary Fig. S6). Analysis of tumor status at diagnosis versus time of enrollment confirmed that most participants had developed metastatic disease by the time of enrollment (Table 1), although 20% of participants had no evidence of disease (NED), highlighting the wide range of disease states of enrolling participants.

Baseline performance scores were relatively high in both pediatric and adult patients (Table 1; Supplementary Fig. S7A). Patients with chordoma reported the highest general health at baseline, with few respondents reporting their health as poor (Supplementary Fig. S7B). Most participants <20 years old were in a healthy weight range (Supplementary Fig. S7C), whereas most adults ≥20 years old were overweight or obese (Supplementary Fig. S7D). However, the proportion of overweight/obese participants was similar to U.S. obesity trends (Supplementary Fig. S7E).

### Social/Environmental Variables

The most frequent occupations reported were management, office/administration support, and business/financial operations (Supplementary Fig. S8A) and education data showed that 75% of adults over 21 completed college or graduate school (Supplementary Fig. S8B). Collection of substance use data from clinic evaluations and by self-report of tobacco use and exposure showed low levels of tobacco exposure and recreational drug use (Supplementary Fig. S8C and S8D).

### Other Health Issues

Self-reported data of other health issues experienced by participants demonstrated that allergies to medicines and environmental factors were widely reported in all groups (Supplementary Fig. S9). Anxiety and depression were reported in all groups except family members who do not carry a known germline mutation. Hyperthyroidism or hypothyroidism was reported by participants with MTC, ACC, sdGIST, and NEN. Relatively more participants with ACC and NEN reported asthma and high blood pressure. Gastrointestinal disorders, including colitis/Crohn disease/inflammatory bowel disease and gastroesophageal reflux disease were reported by 17% of participants with NEN, with the majority being diagnosed with neuroendocrine tumor (NET) of the gastrointestinal tract. Patients with chordoma reported developmental delays and mental illness, in addition to anxiety and depression. Patients with sdGIST and NEN reported ulcers. For comparison, family members reported common health problems for their age group, such as high cholesterol and diabetes in older individuals.

### Prior Tumor-directed Therapy

At initial diagnosis, most rare tumor patients were treated either with surgery alone, or surgery combined with drug treatment (Supplementary Fig. S10A). NEN was the only group with a substantial number of patients receiving medical therapy without surgery at initial diagnosis. Surgery was the most common treatment across tumor types, with 88% of patients undergoing at least one surgery (Supplementary Fig. S10B). Cytotoxic chemotherapy was more common for ACC, whereas targeted therapy was more common in sdGIST, and radiotherapy and hormone therapy were more common in NEN.

### PRO Results for Anxiety, Depression, and Pain

PROMIS measures assessing depression, anxiety, and pain interference were completed by 114 adult patients (≥18 years) at baseline. Across patient groups, mean T-scores were within normal limits for depression, anxiety, and pain interference (Table 2). For groups with at least 10 respondents, the percentage of respondents reporting CS anxiety ranged from 20% to 35%. Fewer respondents reported CS depression, with ACC (17%) demonstrating the highest percentage of CS depression. We assessed depression and anxiety scores in participants further out from diagnosis compared with newly diagnosed and show that while anxiety and depression trend lower further from initial diagnosis, there were still participants with CS anxiety and/or depression up to 8 years later (Supplementary Fig. S11). CS pain interference ranged from 14% to 22% of respondents for groups with ≥10 respondents.

**TABLE 2:** Adult self-report of PROMIS data

	Total Responses		CS^a^ Responses	
PROMIS Measure/Tumor	*n*	Mean (SD)	*n*	%
Anxiety				
ACC	23	55.6 (8.8)	8	34.8
Chordoma	9	55.3 (8.5)	3	33.3
GIST	20	54.9 (7.1)	4	20.0
MTC	8	56.4 (9.2)	4	50.0
NEN	32	55.4 (7.7)	7	21.9
Other	22	50.9 (11.7)	5	22.7
Depression				
ACC	23	49.7 (9.6)	4	17.4
Chordoma	9	48.5 (9.2)	1	11.1
GIST	20	47.8 (8.0)	1	5.0
MTC	8	49.2 (8.0)	0	0
NEN	32	50.0 (8.6)	3	9.4
Other	22	46.7 (9.7)	3	13.6
Pain interference				
ACC	23	51.1 (9.8)	5	21.7
Chordoma	9	49.2 (8.7)	1	11.1
GIST	20	51.7 (8.8)	3	15.0
MTC	8	57.4 (6.6)	3	37.5
NEN	32	52.7 (8.6)	5	15.6
Other	22	48.2 (9.8)	3	13.6

NOTE: T-scores are compared with a mean of 50, SD of 10.

Abbreviation: CS: clinically significant.

^a^CS range is ≥60.

### Tumors in First-degree Relatives

Using genetic counseling interviews and self-reported family tumor cases, we found that many participants had a first-degree relative with a tumor diagnosis, ranging from 33% to 57% depending on tumor type (Table 1).

### Tumor and Germline Mutations

Of 180 participants with rare tumors, 147 had at least one pathology sample available for analysis. For 117 participants, successful panel sequencing was performed on 130 total tumor samples. Of the remaining 30 participants, all available samples failed sequencing for a variety of reasons (e.g., low tumor content or poor DNA quality). Analyzing the age of submitted formalin-fixed paraffin-embedded (FFPE) samples (Supplementary Fig. S12), newer samples tended to be more successful; however, sequencing could be successful in older samples of up to approximately 10 years, and more recent samples could also fail.

**FIGURE 2:**
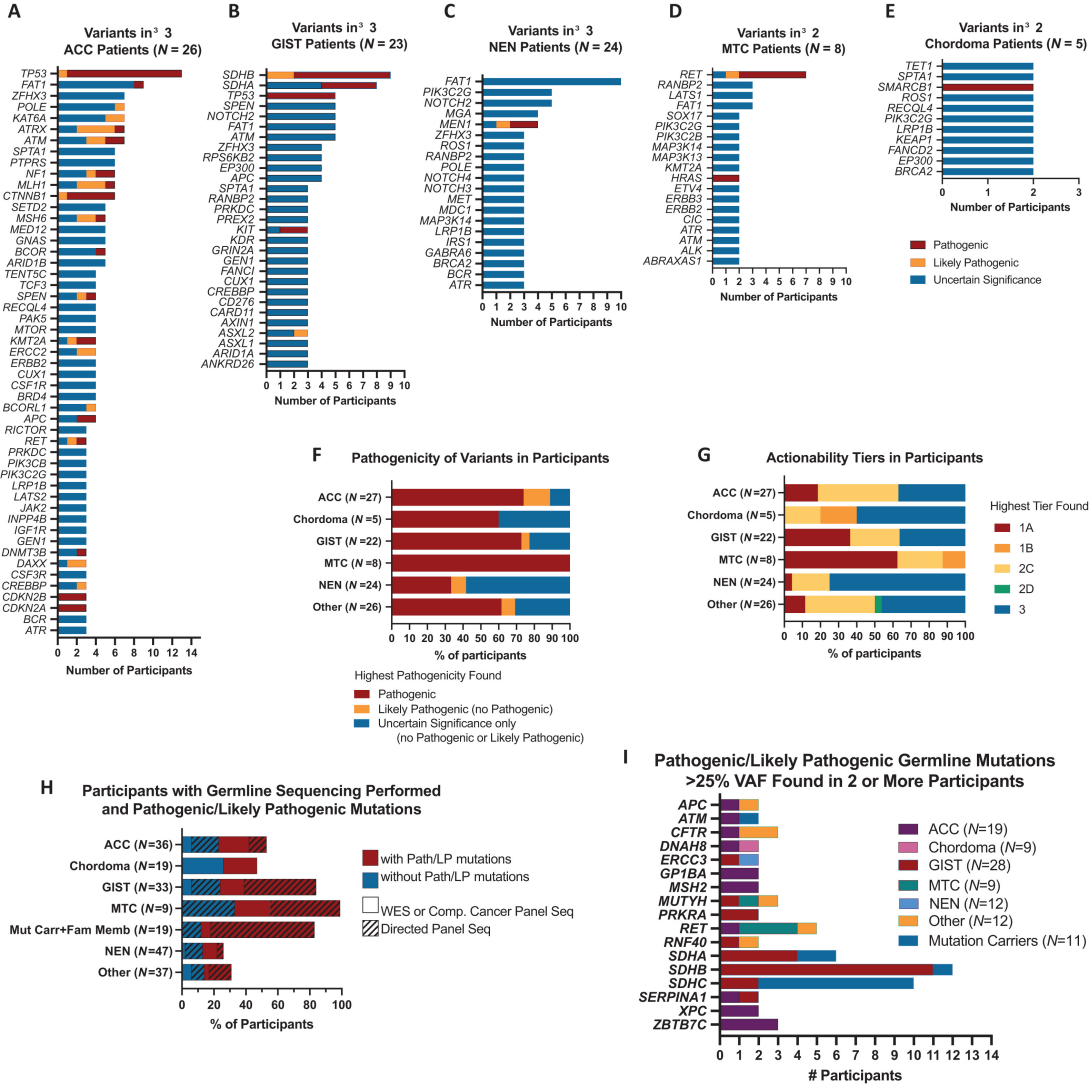
Variant genes found in 3 or more patients for ACC, *N* = 27 (**A**), GIST, *N* = 22 (**B**), and NEN, *N* = 24 (**C**). Variant genes found in 2 or more patients for MTC, *N* = 8 (**D**) and chordoma, *N* = 5 (**E**). TSO500 gene panel sequencing identified pathogenic or likely pathogenic mutations for most tumors (**F**), with NEN and chordoma showing the lowest percentage of pathogenic mutations identified. ACC and MTC tumor analysis identified the highest percentage of pathogenic mutations. **G,** sdGIST and MTC had the most actionable mutations in Tier 1A. Germline sequencing on participants was performed using directed variant, single gene, or small gene panels (<100 genes; hatched bars) or using comprehensive large gene panels or WES (solid bars; **H**). The proportion of Path/LP mutations identified (red) varied by tumor type. **I,** The most common Path/LP germline mutations found were *SDHA/B/C,* in participants with sdGIST and their family members, and *RET*, in an ACC, three MTCs, and a pheochromocytoma.

TSO500 assay results for 86 participants in the largest tumor cohorts are shown in Fig 2. In ACCs, Path/LP variants were found in *TP53, CTNNB1, ATRX, MLH1*, *ATM, CDKN2A/CDKN2B, KMT2A,* and *MSH6* in 3 or more patients (Fig. 2A), but only 63% of patients with ACC had at least one of these mutations and 10 participants had Path/LP variants in genes found in fewer than 3 patients, demonstrating the molecular heterogeneity of this ACC cohort. Most sdGISTs had Path/LP mutations in one of the SDH subunit genes (*SDHA N* = 4, *SDHB N* = 8, *SDHC N* = 2; Fig. 2B) or hypermethylation of the *SDHC* promoter (epimutants). Consistent with previous reports, only female epimutants were identified (29)*.* NENs were molecularly diverse (Fig. 2C), with Path/LP variants in *MEN1* detected in tumors from 3 participants. Two of these patients do not have germline *MEN1* mutations. The third has not completed germline testing for *MEN1* mutations, to date. Pathogenic *TP53* variants were found in 2 NEN participants and all other Path/LP variants detected (*N* = 14) were found in a single participant. For 14 NEN participants (58%), no Path/LP variants were found. Path/LP variants in *RET* were found in 6 out of 8 patients with MTC (Fig. 2D), with 3 having germline mutations indicative of MEN type 2 (30). *HRAS* mutations were found in patients without a *RET* Path/LP variant. Pathogenic *SMARCB1* variants were found in 2 patients with chordoma (Fig. 2E). Overall, 19% of TSO500 genes had a Path/LP variant identified (Supplementary Table S1) and 71% of participants tested had at least one Path/LP variant (Fig. 2F) with the fewest found in NEN. Actionable mutations (Tier 1A/1B/2C/2D) were found in 55% of participants (Fig. 2G).

Of the first 200 participants, 102 either had record of previous germline mutation testing or were evaluated for GCPS at NIHCC. Germline mutation testing of participants ranged from examining a specific variant in family members to genome-wide WES (Fig. 2H; Supplementary Table S2). *SDHB*, *SDHC*, and *SDHA* were the most common germline Path/LP variants identified (Fig. 2I), found in participants with sdGIST and their relatives. Path/LP variants in *RET* were identified in 5 participants with different tumor types. Path/LP variants were also found in 3 participants for *CFTR, MUTYH*, and *ZBTB7C.* Of note, six out of 12 GCPS cases were newly diagnosed on NHRST. Of 102 participants with germline data available, 10 had germline VUS identified without any pathogenic germline variant.

### N-of-1 Patients

**FIGURE 3:**
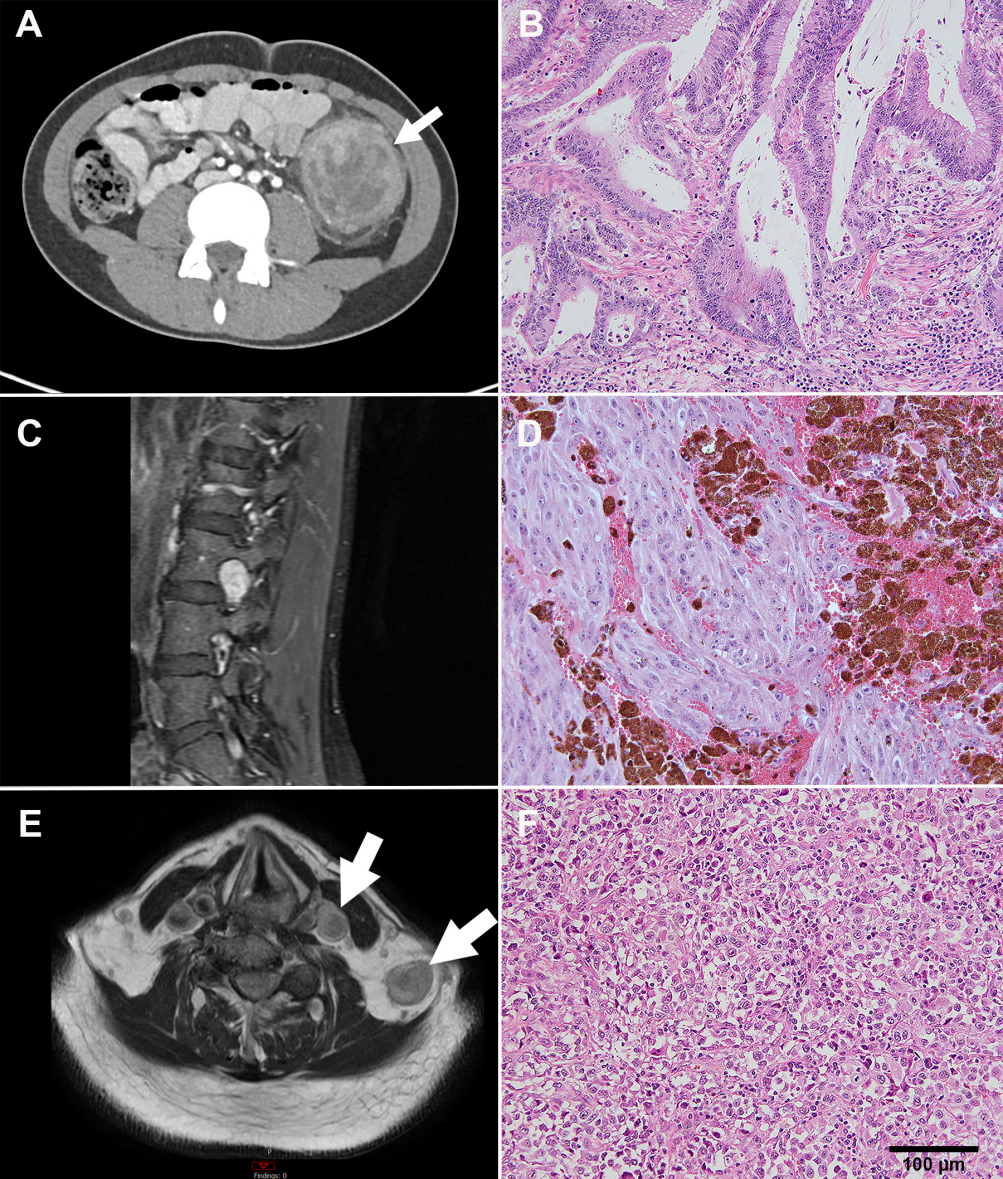
Imaging and histology for 3 highlighted patients. **A,** Axial CT image of the abdomen showing a mass in the descending colon (arrow) in a 15-year-old male diagnosed with poorly differentiated adenocarcinoma. **B,** Histology shows high dysplasia and stromal microinvasion. **C,** MRI of the lumbar spine in a 36-year-old female diagnosed with malignant melanotic schwannoma shows oval-shaped mass in left neural foramen of L3-L4 with avid enhancement on sagittal T1-weighted fat-suppressed image (arrow). Histology (**D**) shows pigmented spindle and epithelioid cells. **E,** MRI displays two left supraclavicular masses on T1-weighted image in a 75-year-old male with metastatic poorly differentiated carcinoma of unknown primary in the neck. **F,** Histology shows epithelioid tumor cells. Scale bar = 100 µm and is the same for B, D, and F.

**FIGURE 4:**
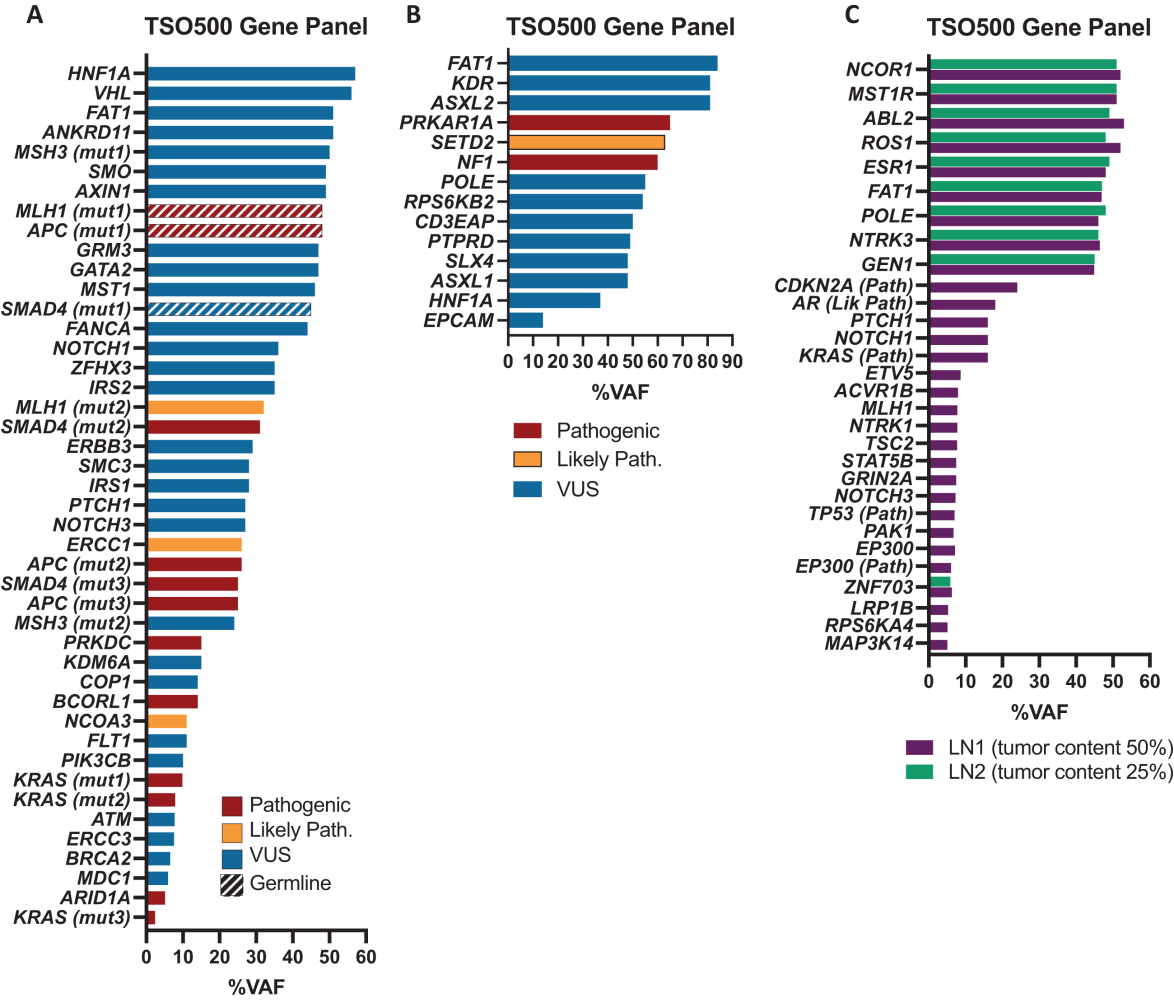
Molecular analysis for 3 highlighted patients. **A,** Path (red), LP (gold), and VUS (Blue) variants identified by TSO500 in colon adenocarcinoma from 15-year-old male. Pathogenic germline mutations were identified in *MLH1* (c.156del p.Glu53Argfs*4) and *APC* (c.3329C>A, p.Ser1110*) and VUS in *SMAD4 (*c.947A>G, p.Asn316Ser; hatched bars). **B,** Variants identified by TSO500 in a malignant melanotic schwannoma from a 36-year-old female, with Path mutations in *NF1* (c.276dupA, p.C93fs*14) and *PRKAR1A* (c.207_208delGA, p.K70fs*11) and LP variant in *SETD2* (c.4405dupA, p.M1469fs*6)*.***C,** Variants identified in two lymph node metastases collected 1 year apart from a carcinoma of unknown primary in a 75-year-old male (purple collected first, green collected second). VUS with higher percentage VAF (>45%) were identified in both samples (*NCOR1, MST1R, ABL2, ROS1, ESR1, FAT1, POLE, NTRK3*, and *GEN1)*, as well as low VAF (∼6%) VUS in *ZNF703*. Pathogenicity of variants is indicated in *y*-axis label.

Of 25 participants enrolled with a diagnosis only represented once in our cohort, three are presented in clinical (Fig. 3) and molecular (Fig. 4) detail, representing three age groups enrolled on NHRST (<18, 18–39, and ≥40 years old).

A pediatric patient was diagnosed with poorly differentiated adenocarcinoma of the descending colon at age 15. He presented with symptoms of abdominal pain, 30-pound weight loss, and nausea/vomiting. Imaging showed a descending colon mass (Fig. 3A). Pathology showed multiple colonic tubular adenomas, and focal adenocarcinoma (Fig. 3B). The patient was found to have pathogenic germline mutations in *MLH1* and *APC* and VUS (strong evidence of pathogenicity) in *SMAD4* (Fig. 4A). Molecular tumor testing (Fig. 4A) revealed pathogenic mutations with low VAF in *KRAS, ARID1A, BCORL1,* and *PRKDC*. Low VAF LP variants were found in *NCOA3* and *ERCC1.* Multiple additional mutations were identified in *APC, MLH1,* and *SMAD4.* The patient was treated with surgery followed by capecitabine/oxaliplatin for seven cycles and subsequent prophylactic total colectomy. He continues to be followed according to guidelines for Lynch syndrome and familial adenomatous polyposis.

A 36-year-old female presented with back pain and was diagnosed with malignant melanotic schwannoma of the lumbar spine. MRI showed a mass in the left neural foramen of L3-L4 (Fig. 3C). Pathologic examination by IHC showed positive S100*,* HMB45*,* Melan-A stains with pigmented spindle and epithelioid cells (Fig. 3D) and psammoma bodies suggestive of Carney Complex (31). Germline mutation of *PRKAR1A* was negative, although a pathogenic *PRKAR1A* mutation was identified on tumor sequencing and mosaicism for germline *PRKAR1A* mutation could not be excluded. In addition, a somatic pathogenic variant in *NF1* and LP variant in *SETD2* were identified (Fig. 4B). She was treated with surgery, photon radiotherapy, and nivolumab/ipilimumab, but died from disease 2 years after diagnosis.

A 75-year-old male presenting with left neck pain was diagnosed with metastatic poorly differentiated carcinoma of unknown primary. MRI scan showed a left supraclavicular mass (Fig. 3E) and avid 2[18F]fluoro-2-deoxy-D-glucose uptake on PET scan. Biopsy demonstrated a malignant epithelioid tumor (Fig. 3F) positive for keratins (AE1/AE3) and weakly positive for PAX8. Because PAX8 is a common marker of renal cell carcinoma (32), the carcinoma was suspected to be metastatic renal carcinoma; however, no renal primary tumor could be identified. Two metastases to lymph nodes collected 1 year apart were sequenced (Fig. 4C). Path/LP mutations with low VAF were identified in *KRAS, TP53, AR, CDKN2A,* and *EP300* in the first sample, but not the second sample, likely due to lower tumor content of the second sample. VUS with greater percentage VAF (>45%) were identified in both. He was treated with photon radiotherapy and surgical resection and is alive 3 years after diagnosis.

## Discussion

We developed the NHRST to advance rare tumor research and ultimately improve patient outcomes through comprehensive study of rare solid tumors while engaging patients, advocates, and researchers. We were able to successfully enroll participants with rare tumors and collect comprehensive standardized clinical information on 98% of participants, self-reported PRO from 74% of participants, and tumor and/or germline biospecimens from 87% of participants. Analysis of the first 197 participants (out of 200 enrolled) of our ambitious study revealed several findings, which serve to inform the future of our study and possibly other rare tumor efforts.

Our study design allowed for enrollment in person at the NIHCC or remotely from the participants’ homes. Study enrollment began the year before the COVID-19 pandemic and remote enrollment allowed continued study participation and study feasibility through the pandemic. This may thus be an efficient approach in future studies.

Despite broad eligibility of any patient with a rare solid tumor, our cohort was not balanced in that we enrolled more participants from the U.S. East Coast closer to the NIHCC in Bethesda, MD, more females than males, and predominately white, non-Hispanic, more highly educated participants. To increase geographic diversity and recruitment of patients from the Southwest and Western United States, we are expanding the study to partner institutions. Several rare tumors in NHRST such as ACC and sdGIST have reported higher incidence in females (29, 33, 34). However, while most of our MTC participants were female (89%), a larger study showed little sex bias in sporadic and hereditary MTC (35). While participants are predominantly White and non-Hispanic, self-reported data on ancestry demonstrated contributions of different races to biospecimen genetic background. We found that reported race and ethnicity in the medical record was inconsistent with self-reported data, which were more detailed, demonstrating the value of self-reporting to reflect race and ethnicity accurately. Our participants showed high levels of education, with management, administrative support, and business/financial operations being the most reported occupations. Previous research has shown that education and income levels can affect diagnosis and progression of disease (36, 37). Recruitment to NHRST relies on referrals by physicians, and on patients finding our study through internet searches or interactions with advocacy groups, which may require higher levels of computer and medical literacy. Sex, race, and ethnicity can impact tumorigenesis and response to treatments both biologically (38) and socioeconomically (39, 40). We recognize that enrollment bias in these factors may limit the relevance of our findings to diverse patient populations and are aiming to develop targeted recruitment strategies for underrepresented populations.

Using a histology-agnostic approach, we enrolled participants with 35 different types of rare solid tumors. NIHCC infrastructure and collaboration among pediatric and adult oncologists facilitated enrollment of both pediatric and adult rare tumor patients, with 34% of participants being AYA, a population of unmet need for whom tumor incidence is increasing (41). We were also able to enroll pediatric participants with tumors diagnosed commonly in adults, but rarely in children, such as colorectal cancer, which is increasing in incidence in younger individuals (42). While any patient with a rare solid tumor was eligible, we enrolled five tumor types (NET, ACC, sdGIST, chordoma, MTC) with 9–47 participants per group. These tumors are the subject of strong research interests at NIHCC, including NIH rare tumor clinics for sdGIST, MTC, and chordoma, and strong collaborations with advocacy (43–45). It is thus feasible to enroll cohorts of patients with specific very rare tumor types. Importantly, most of our participants were not newly diagnosed, but enrolled several years after diagnosis when most participants had metastatic disease. This may reflect the large unmet need for this population where interventional trials are frequently not available and curative approaches are lacking. Recurrent disease after standard upfront therapy may also be the disease status for which development of external controls may be most needed, as novel interventional agents are typically first tested in the relapsed/refractory setting. This highlights the potential of NHRST to illuminate the natural history of recurrent disease and long-term survival and develop robust external controls for intervention trials.

Although we are collecting and extracting retrospective data on prior medical history and treatment, as well as following GCPS for development of tumors, it would be ideal to robustly characterize the clinical course of all very rare tumors of unmet need from diagnosis throughout the disease trajectory. Rapid progress will benefit from dedicated effort by additional “rare tumor champions” for specific rare tumors working with clinicians, researchers, and advocacy to develop additional patient cohorts. NCI's Childhood Cancer Data Initiative (CCDI) in collaboration with the Children's Oncology Group has established the Molecular Characterization Initiative (46), which provides state-of-the-art clinical molecular profiling and collection of clinical information focused on children and young adults with newly diagnosed malignancies including rare malignancies. The NCI CCDI is currently exploring the possibility of a CCDI-coordinated national very rare cancer longitudinal study (46) with participation by multiple sites and consortia.

In addition to information included in the medical record such as imaging, pathology, and medical history, we included PROs and comprehensive, standardized self-reported family and medical history. Although we had significantly fewer respondents completing self-report forms in the field group, we demonstrated feasibility of remote PRO administration to gather data from participants unable to travel due to illness, or other circumstances. PROs can be used to characterize quality of life relative to disease status, effectiveness of therapies, adverse effects of drugs, disease progression (47), and comparing patients with reference populations (e.g., comparing chordoma with other skull-based tumors). Patients with rare and potentially hereditary tumors are at risk for psychologic problems throughout life. Indeed, we found that anxiety and depression can remain CS long after initial diagnosis. Although there are many potential benefits to obtaining PROs from both in-clinic and field cohorts, we also acknowledge several limitations. First, our sample is potentially biased toward those with progressive disease who may be more likely to reach out to the NIH, enroll on the study, and report CS symptoms. Notably, 63% of our cohort had metastatic disease at enrollment. However, because many of our patients were further out from diagnosis at the time of enrollment, the impact of disease status on PROs at the time of diagnosis, is largely unavailable to comment on. In contrast, for field cohort participants, progressive disease may lead to difficulty completing PROs without in-person assistance. Similarly, this study provides insight into depression, pain, and anxiety symptoms at a single timepoint, and does not consider social-emotional history that preceded the cancer diagnosis, or life events that may contribute to elevated mental health symptoms at the time of PRO administration. Although administering PROs remotely is advantageous for patients unable to travel, we note anecdotally that patients completing PROs during visits to NIHCC ask clinical staff for clarification of questions, an opportunity lacking for remote participants. In the case of PROs administered to children, it is unknown how parental interactions in the remote setting might bias how children respond to questions. These confounding factors must be considered when weighing benefits of remote PRO administration in developing external control groups. Future strategies to increase patient engagement and completion of PRO measures, may include offering PROMIS forms in languages other than English and identification of challenges to form completion. In addition, we plan to conduct in-depth, longitudinal analysis of PROs by tumor type to include correlation of PRO data with measures of disease severity.

Broad collection of health-related data allows for comparisons between participants and other patient cohorts or the general population. Although 29% of measured adult participants were obese, this was less than 2017–2018 U.S. population data from NHAHES (42% obese). Our cohort had a bias toward White college-educated women, who typically have lower rates of obesity (48). Therefore, more data and detailed analyses are needed to make BMI comparisons by demographic and tumor type. When looking at participant reported health issues, some were common across all groups (e.g., allergies) likely reflecting the prevalence of these issues in the United States, whereas others were more frequent in certain tumor cohorts. Some health issues associated with specific tumor types likely reflect effects of tumor or treatment on the body (e.g., hypothyroidism or hyperthyroidism resulting from treatment in MTC or high blood pressure due to hormone disruption in ACC), whereas others (e.g., developmental delay in chordoma) could suggest genetic or developmental factors co-occurring with tumor risk or effects of treatment, particularly in developing children.

The molecular drivers of tumorigenesis are unknown for many rare tumors. To identify new targets for therapeutic intervention we analyzed genomic results from tumor biospecimens. *TP53* and *CTNNB1* are well-established ACC drivers (49–51). Path/LP variants found in ACC are involved in negative regulation of cell cycle and cell-cycle arrest (*ATM, ATRX, CDKN2A, CDKN2B, CTNNB1, MSH6,* and *TP53*), chromatin remodeling and organization (*ATM, ATRX, CDKN2A, CTNNB1,* and *KMT2A*), DNA repair and response to DNA damage (*ATM, ATRX, MLH1, MSH6,* and *TP53*), and intrinsic apoptosis (*ATM, MLH1, MSH6,* and *TP53*). While most GIST carry activating mutations in *KIT* or *PDGFRA* (52), our study focuses on a rare subset, sdGIST, lacking mutations in *KIT* or *PDGFRA*, which are resistant to imatinib and found in younger patients. sdGIST are driven by germline mutations or epigenetic silencing of the SDHC promoter (29, 43). These alterations were also observed in our patient cohort as well as a subset of their relatives. Mutations in *RET,* found in most MTCs in NHRST, are also found in the GCPS MEN2A and MEN2B, and *RET* is being targeted for systemic therapies (53–55). Somatic mutations in *MEN1* have been previously identified in 44% of pancreatic NETs (56) and were found in 13% of our patients with NEN, including 2 with pancreatic NET. In our cohort, very few driver mutations were identified in chordoma, although amplification of brachyury (57) was not examined and only a small number of biospecimens were available for analysis. Loss of INI1 expression, encoded by *SMARCB1*, is characteristic of poorly differentiated chordomas more common in children and young adults (56). In addition to the Path/LP variants, many of the VUSs identified in our cohort are in molecular pathways implicated in rare tumors; studies are ongoing for further characterization of all VUSs.

In a study of 1120 pediatric patients (58), Path/LP germline mutations were found in 8.5% of patients and approximately 7%–14% of pediatric tumors are associated with GCPS (59). A survey of 27,999 adults found that 35.6% of respondents reported cancer in a first-degree relative (60). We found that 46% of participants had a first-degree relative with cancer. Of participants with clinically directed germline sequencing, 65% had at least one Path/LP germline mutation (33% of total cohort), likely due to our initial focus on sdGIST and MEN2A/B. Because many patients had targeted sequencing, rather than WES, the number Path/LP mutations present is likely underestimated. Future studies could identify new GCPS in addition to those we focused on.

## Challenges

Over the course of enrolling and following the first 200 participants, we identified several challenges in the execution of NHRST. First, the COVID pandemic impeded enrollment, particularly for the clinic group, preventing collection of biospecimens and data from physical exams, imaging, and clinical labs. In contrast, although remote participation had many advantages, we found it was less robust for collecting self-reported data. Second, to take advantage of archival tumor samples for longitudinal analysis, we focused our molecular analysis on FFPE samples. In general, we found that sample quality becomes compromised after 5 years, limiting retrospective analyses. Third, in comparing overlapping self-reported and clinician-collected information, we found discrepancies, primarily due to exact phrasing and interpretation of questions. Finally, we have found that focused effort by clinicians and advocates is required to build and study useful cohorts for rare tumors.

## Future Directions

We established a comprehensive, longitudinal natural history study for rare solid tumor types in children and adults, demonstrating how data specific to rare tumor types (e.g., correlated health issues, molecular drivers, and PROs) can be collected in a tumor-agnostic manner. Going forward, we plan to focus on developing interventional trials, increasing representation of minority and underserved populations, building select additional cohorts, and analyzing longitudinal data and comprehensive biospecimen molecular data. Interventional trials could enroll multiple rare tumor types based on mutation status, such as the recently opened trial for *SMARCB1/SMARCA4*-deficient tumors (NCT05286801). Increasing representation may be facilitated by developing specific partnerships with hospitals, advocacy groups, and professional organizations targeting underrepresented groups. Increasing enrollment of very rare solid tumors affecting children and AYA will be achieved by opening NHRST at other institutions and collaborating with other rare tumor efforts. Longitudinal timepoints will be compared to analyze tumor progression and response to treatment. Collected biospecimens are being comprehensively sequenced to interrogate the transcriptome, genome, and epigenome, in addition to the focused gene panel sequencing described here. As we gather data on NHRST, tumor-specific subprotocols will be developed to collect data tailored for use in interventional trials as external controls. Comprehensive longitudinal data gathered will be shared to facilitate collaborative rare tumor research. All data will be made publicly available per requirement of the NCI Cancer Moonshot^SM^. Data presented here are being made available through dbGAP and the NCI Cancer Data Service. Additional data from the study that is analyzed in future publications will be released upon validation in NCI-supported databases with controlled access provided by dbGAP.

## Supplementary Material

Supplementary Tables S1 and S2 Table S1 shows genes assayed in the TSO500 panel. Table S2 shows the sources of germline data for individual subjects.

Supplementary Fig 1 Geographic distribution of participants by age group.

Supplementary Fig 2 Age at diagnosis.

Supplementary Fig 3 Race and ancestry of participants.

Supplementary Fig 4 Distribution of enrollees participating remotely (field; red) or traveling to NIHCC (clinic; blue), located in Bethesda, MD.

Supplementary Fig 5 Protocol form completion.

Supplementary Fig 6 Time from diagnosis to enrollment by tumor type.

Supplementary Fig 7 Performance scores, self-reported general health rating, and BMI.

Supplementary Fig 8 Example of social and environmental characteristics of the NHRST cohort.

Supplementary Fig 9 Other health issues reported in 3 or more participants out of 115 respondents by tumor type.

Supplementary Fig 10 Tumor-directed treatments.

Supplementary Fig 11 Anxiety and depression over time.

Supplementary Fig 12 Success of TSO500 panel sequencing depending on age of formalin-fixed paraffin-embedded (FFPE) tissue.
