# Supplementary material for: Longitudinal Natural History Study of Children and Adults with Rare Solid Tumors: Initial Results for First 200 Participants
Source: Cancer Res Commun. 2023 Dec 6;3(12):2468–82. doi: 10.1158/2767-9764.CRC-23-0247 (PMC10699159; doi:10.1158/2767-9764.CRC-23-0247)
Supplement: Supplementary Fig 1 — Geographic distribution of participants by age group. [file crc-23-0247-s02.pdf]

SUPPLEMENTAL FIG 1: Distribution of 197 participants in the first 200 enrolled by age group

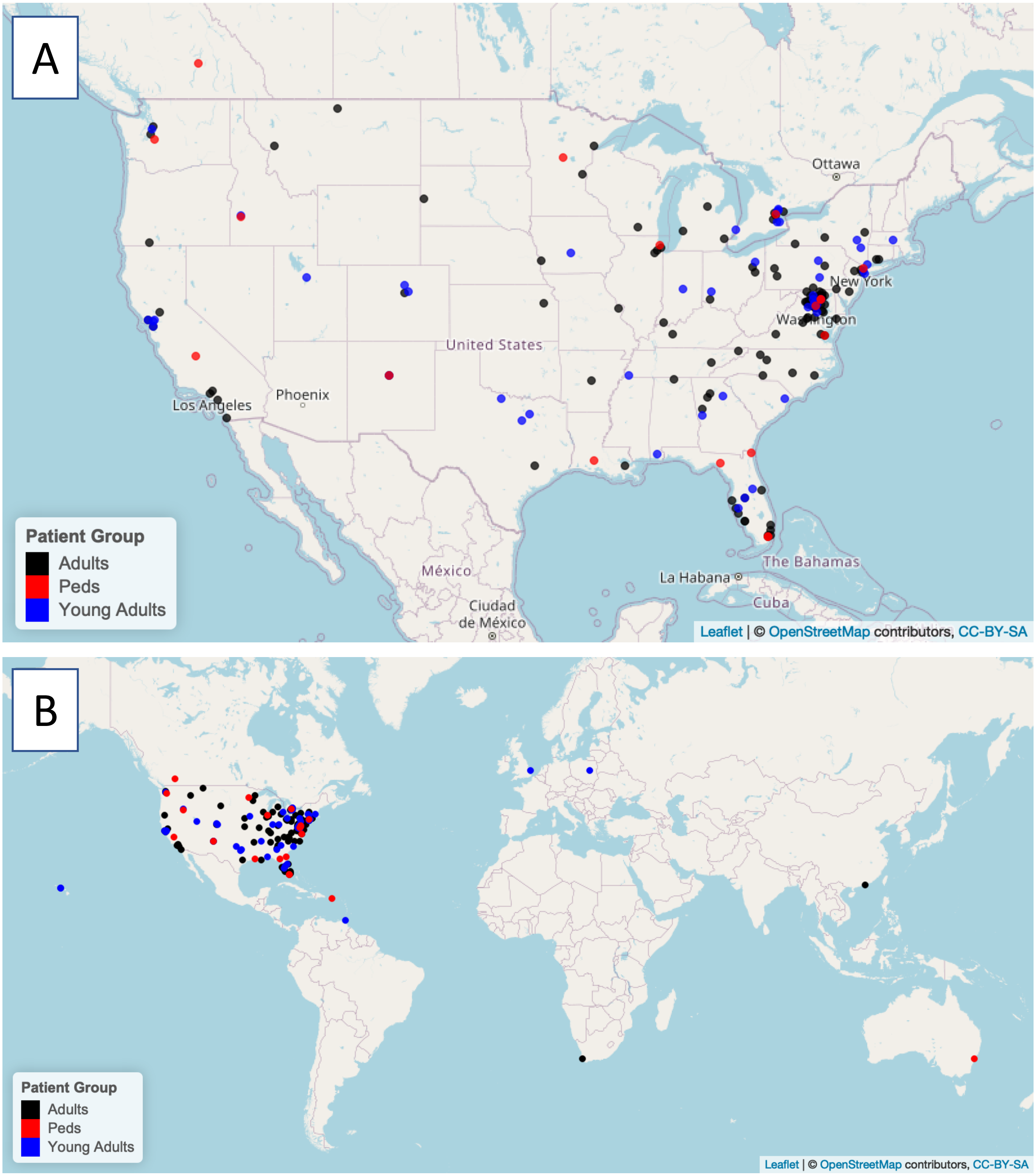

Supplemental Figure 1: Geographic distribution of participants by age group. (A) Distribution of adult (≥40 years old; black), pediatric (<18 years old; red), and young adult (≥ 18 and <40 years old; blue) participants in 46 US states. (B) International distribution of adult (≥40 years old; black), pediatric (<18 years old; red), and young adult (≥ 18 and <40 years old; blue) participants in 9 countries spread across 6 continents.
