# Supplementary material for: Longitudinal Natural History Study of Children and Adults with Rare Solid Tumors: Initial Results for First 200 Participants
Source: Cancer Res Commun. 2023 Dec 6;3(12):2468–82. doi: 10.1158/2767-9764.CRC-23-0247 (PMC10699159; doi:10.1158/2767-9764.CRC-23-0247)
Supplement: Supplementary Fig 2 — Age at diagnosis. [file crc-23-0247-s03.pdf]

SUPPLEMENTAL FIG 2: Age at diagnosis for 180 participants with rare tumors

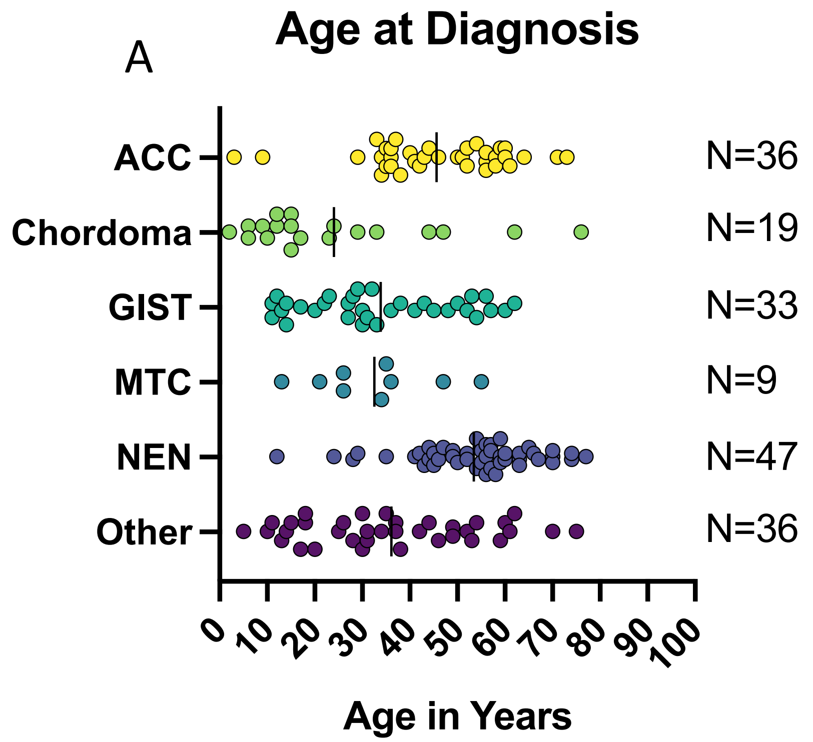

Supplemental Figure 2: Age at diagnosis. Distribution of ages at diagnosis in years (x-axis) by tumor type (y-axis), with NEN showing the oldest average age and chordoma the youngest. Line indicates mean.
