# Supplementary material for: Longitudinal Natural History Study of Children and Adults with Rare Solid Tumors: Initial Results for First 200 Participants
Source: Cancer Res Commun. 2023 Dec 6;3(12):2468–82. doi: 10.1158/2767-9764.CRC-23-0247 (PMC10699159; doi:10.1158/2767-9764.CRC-23-0247)
Supplement: Supplementary Fig 3 — Race and ancestry of participants. [file crc-23-0247-s04.pdf]

SUPPLEMENTAL FIG 3: Race and ancestry of 197 participants

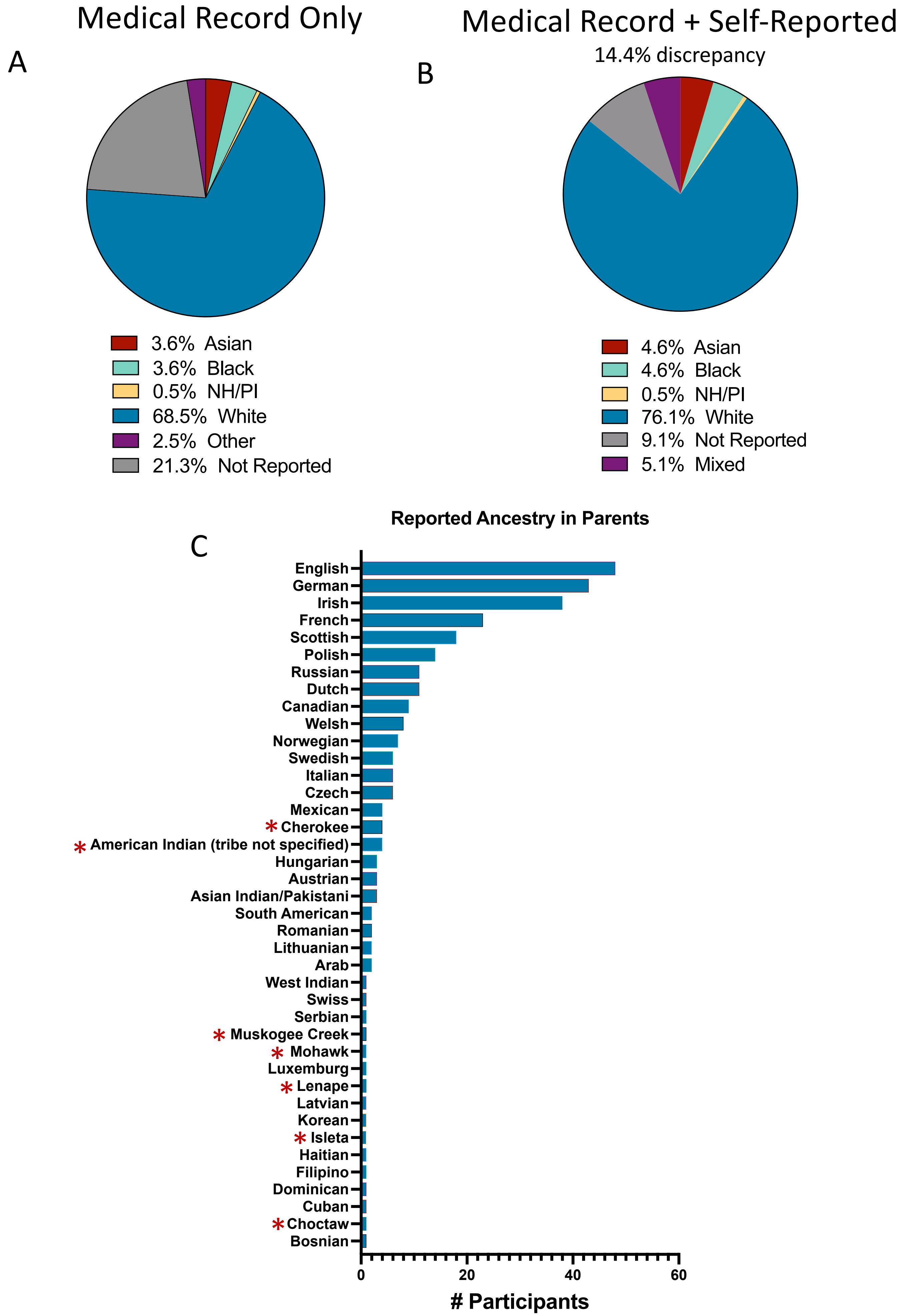

Supplemental Figure 3: Race and ancestry of participants. Race data collected from medical records (A) and self-reported data. Participants were reported as Asian (red), Black (green), Native Hawaiian/Pacific Islander (NH/PI; gold), White (blue), and Other or Mixed (purple). No participants identified as American Indian/Alaska Native. When analyzing the combination of medical records and self-reported data (B) self-reported data is preferentially used when there was a discrepancy (14.4%). (C) Self-reported data on parent ancestry with the number of participants (x-axis) reporting a particular ancestry (y-axis) in their mother or father, highlighting representation of minority racial backgrounds, including American Indian/Alaska Native ancestry (indicated by star on y-axis).
