# Supplementary material for: Longitudinal Natural History Study of Children and Adults with Rare Solid Tumors: Initial Results for First 200 Participants
Source: Cancer Res Commun. 2023 Dec 6;3(12):2468–82. doi: 10.1158/2767-9764.CRC-23-0247 (PMC10699159; doi:10.1158/2767-9764.CRC-23-0247)
Supplement: Supplementary Fig 4 — Distribution of enrollees participating remotely (field; red) or traveling to NIHCC (clinic; blue), located in Bethesda, MD. [file crc-23-0247-s05.pdf]

SUPPLEMENTAL FIG 4: Geographic distribution of clinic and field cohort

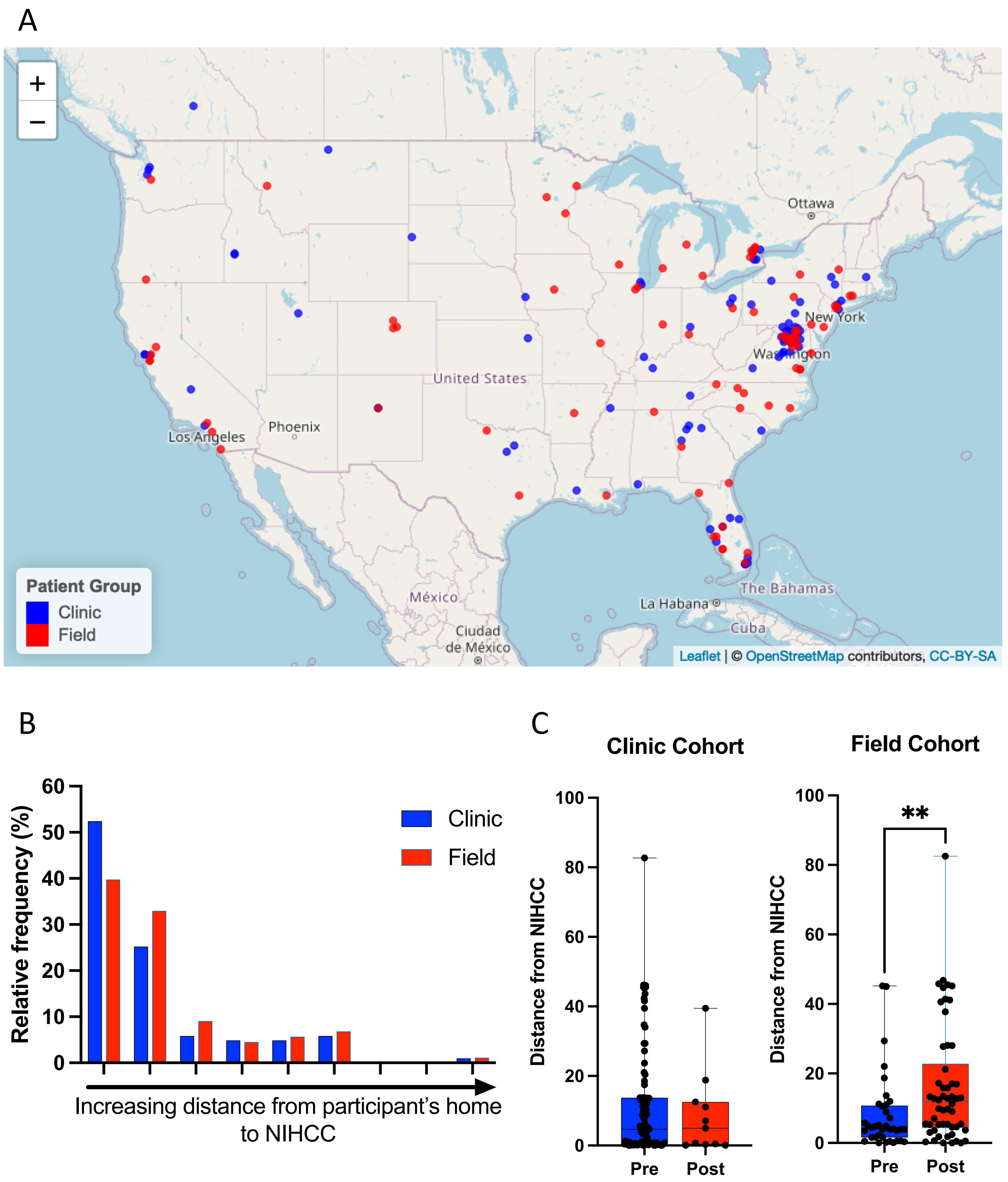

Supplemental Figure 4: Distribution of enrollees participating remotely (field; red) or traveling to NIHCC (clinic; blue), located in Bethesda, MD (A). The majority of participants are concentrated in the area of NIHCC (A), but comparison of field (red) and clinic (blue) groups showed no significant difference (Mann Whitney two-tailed test, P=0.11). Pandemic restrictions starting in March 2020 reduced the number of participants enrolling in the clinic group, but there were no significant differences how far away they lived from NIHCC (Mann Whitney two-tailed test, P=0.53) (B); however there was an increased enrollment of field group participants and a significant increase how far they lived from NIHCC (Mann Whitney two-tailed test, P=0.0046) (C). Distance units are $\sqrt{degrees\_lat^2 + degrees\_long^2}$.
