# Supplementary material for: Longitudinal Natural History Study of Children and Adults with Rare Solid Tumors: Initial Results for First 200 Participants
Source: Cancer Res Commun. 2023 Dec 6;3(12):2468–82. doi: 10.1158/2767-9764.CRC-23-0247 (PMC10699159; doi:10.1158/2767-9764.CRC-23-0247)
Supplement: Supplementary Fig 5 — Protocol form completion. [file crc-23-0247-s06.pdf]

SUPPLEMENTAL FIG 5: Completion of forms by participants and clinical staff

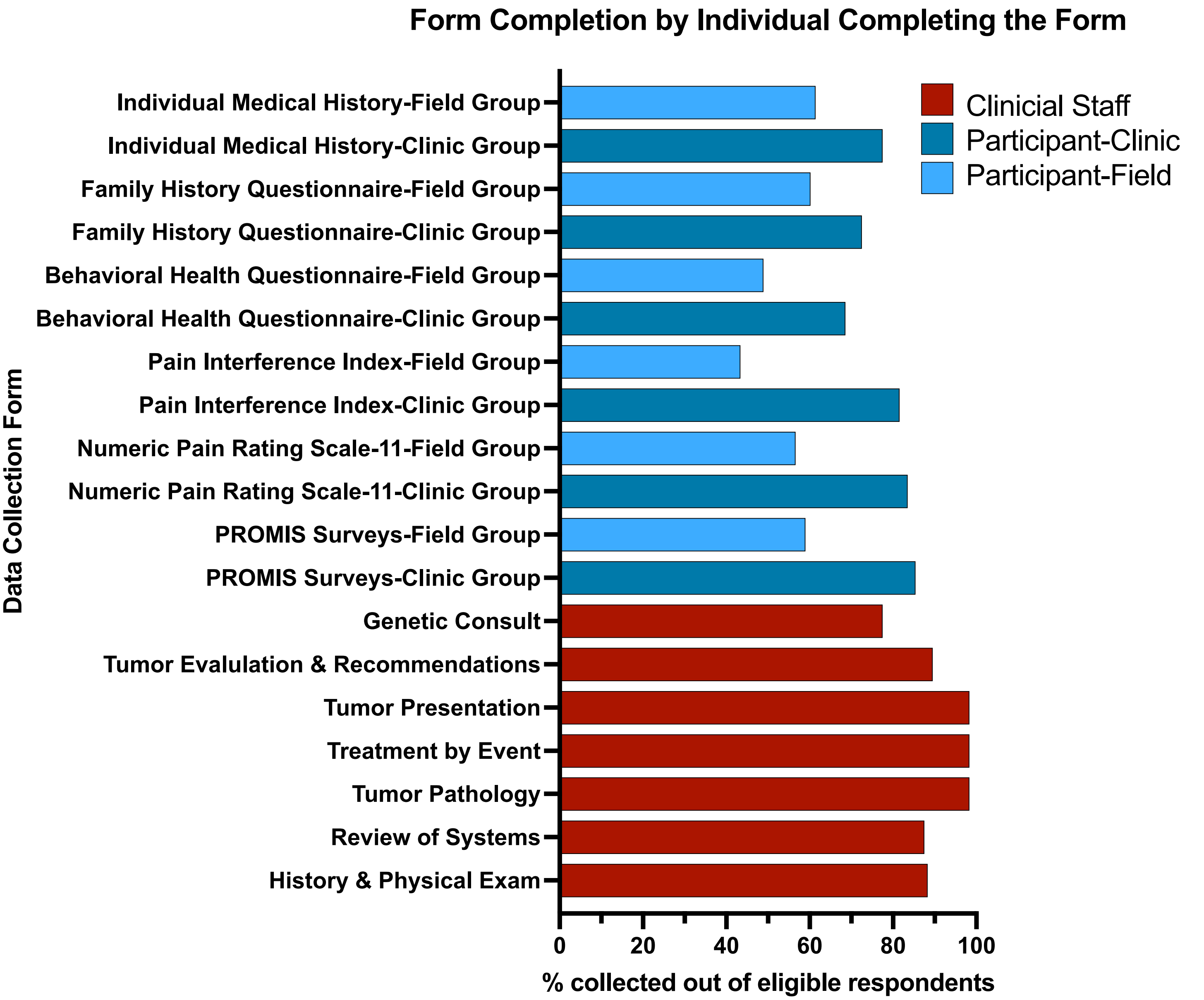

Form	Clinic Group (N=102)	Field Group (N=91)	P value†
Individual Medical History	77.5%	61.4%	0.0178
Family History	72.5%	60.2%	0.0893
Behavioral Health	68.6%	48.9%	0.0075
**Form**	**Clinic Group (N=103*)**	**Field Group (N=83)**	**P value†**
Pain Interference	81.6%	43.4%	<0.0001
Numeric Pain Rating	83.5%	56.6%	<0.0001
PROMIS	85.4%	59.0%	<0.0001

*one non-English speaker was assisted to complete PROs in clinic with help of translator
†two-sided Fisher's exact test

Supplemental Figure 5: Protocol form completion. Forms are indicated by whether they are completed by clinical staff (red), clinic participant (dark blue), or field participant (light blue). The field group completed forms significantly less often than the clinic group (Two-sided Fisher's exact test, $P<0.05$ considered statistically significant). Non-English participants (N=7, all in clinic group) were exempt from completing all forms. Family members without a germline mutation (N=5, all in field group) were exempt from completing the Pain Interference Index, Numeric Pain Rating Scale 11, and PROMIS Surveys. The Tumor Evaluation and Recommendation, Genetic Consult, Review of Systems, and History and Physical Exam form completion was only considered for clinic group participants (N=109).
