# Supplementary material for: Longitudinal Natural History Study of Children and Adults with Rare Solid Tumors: Initial Results for First 200 Participants
Source: Cancer Res Commun. 2023 Dec 6;3(12):2468–82. doi: 10.1158/2767-9764.CRC-23-0247 (PMC10699159; doi:10.1158/2767-9764.CRC-23-0247)
Supplement: Supplementary Fig 6 — Time from diagnosis to enrollment by tumor type. [file crc-23-0247-s07.pdf]

SUPPLEMENTAL FIG 6: Time from initial diagnosis to enrollment for 180 participants with rare tumors

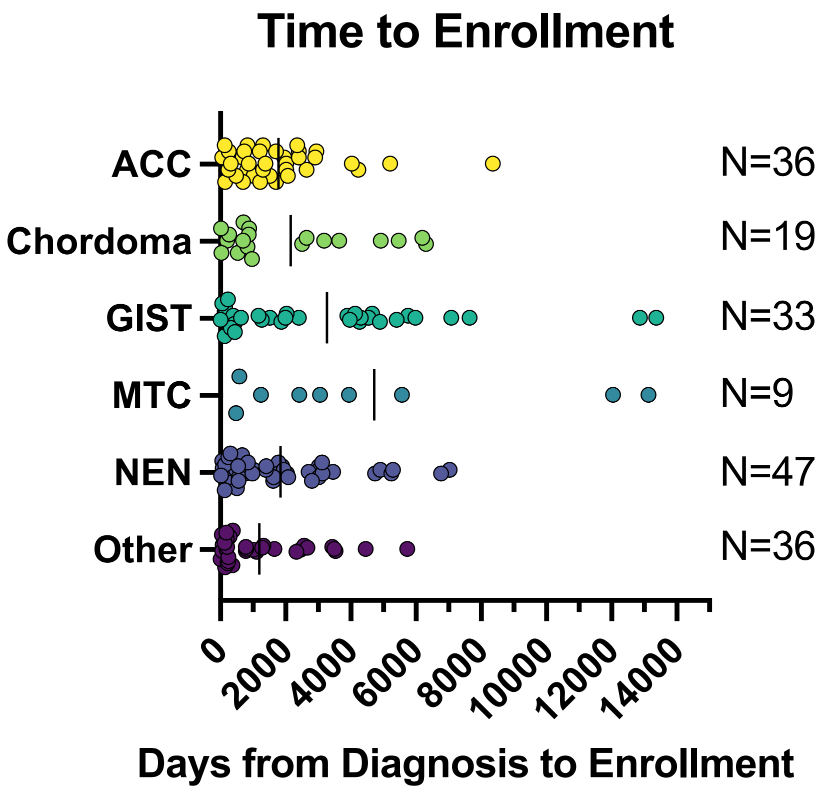

Supplemental Figure 6: Time from diagnosis to enrollment by tumor type. Distribution of the number of days from diagnosis to enrollment (x-axis) for participants by tumor type (y-axis). Line indicates mean.
