# Supplementary material for: Longitudinal Natural History Study of Children and Adults with Rare Solid Tumors: Initial Results for First 200 Participants
Source: Cancer Res Commun. 2023 Dec 6;3(12):2468–82. doi: 10.1158/2767-9764.CRC-23-0247 (PMC10699159; doi:10.1158/2767-9764.CRC-23-0247)
Supplement: Supplementary Fig 7 — Performance scores, self-reported general health rating, and BMI. [file crc-23-0247-s08.pdf]

SUPPLEMENTAL FIG 7: Comparison of performance score and BMI across tumor groups

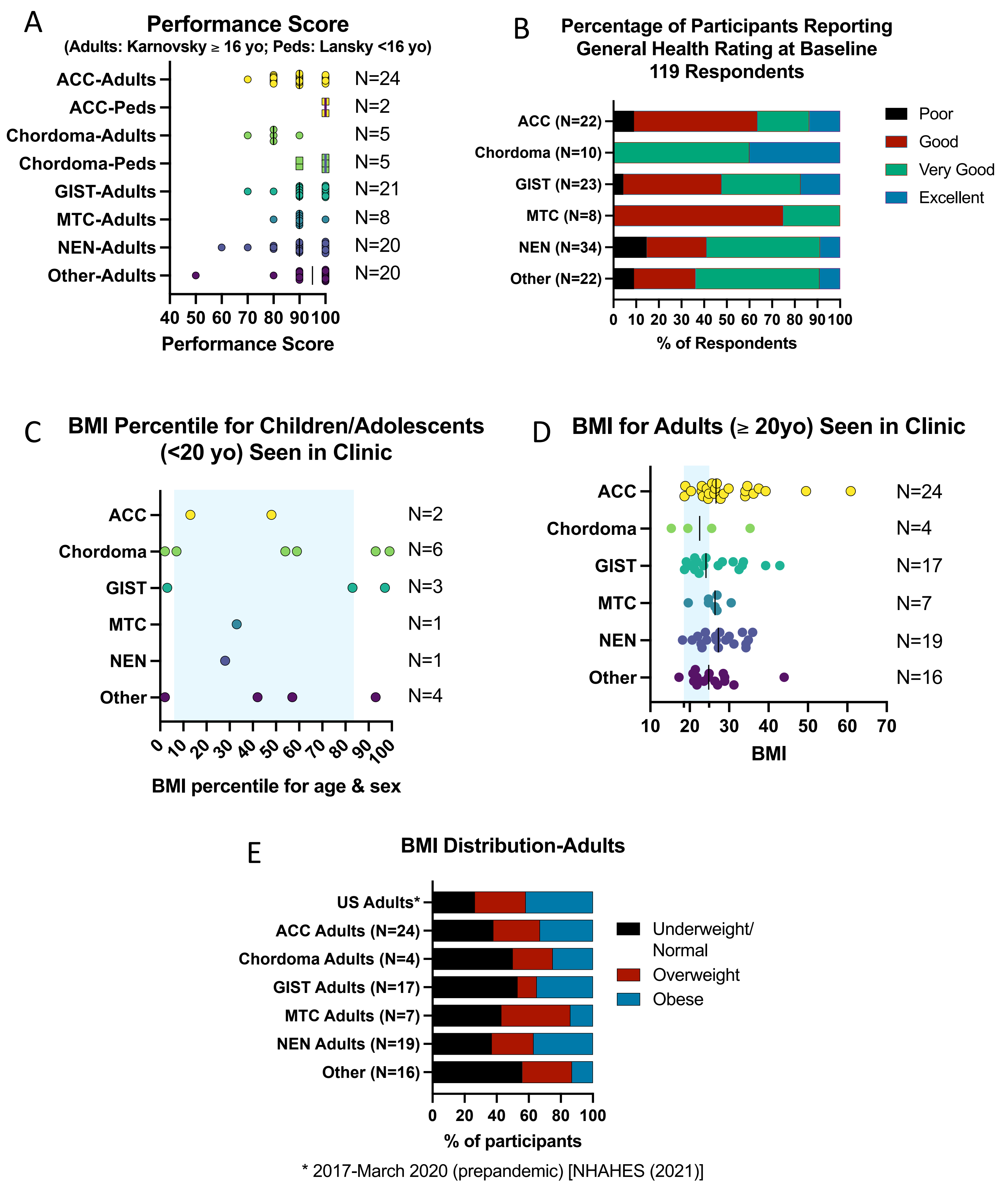

Supplemental Figure 7: Performance scores, self-reported general health rating, and BMI. (A) Performance scores were relatively high in both pediatric and adult patients (line indicates median). (B) The general health rating reported by participants ranging from poor to excellent, with very few participants reporting their overall health as poor. (C) Body mass index (BMI) percentile for children and adolescents seen in clinic <20 years old (blue box indicates normal range). (D) BMI for adults ≥ 20 years old seen in clinic (blue box indicates normal range, line indicates median). (E) BMI distribution of adults categorized as underweight/normal, overweight, and obese compared to US population data collected 2017-2020 prior to the start of the COVID pandemic.
