# Supplementary material for: Longitudinal Natural History Study of Children and Adults with Rare Solid Tumors: Initial Results for First 200 Participants
Source: Cancer Res Commun. 2023 Dec 6;3(12):2468–82. doi: 10.1158/2767-9764.CRC-23-0247 (PMC10699159; doi:10.1158/2767-9764.CRC-23-0247)
Supplement: Supplementary Fig 8 — Example of social and environmental characteristics of the NHRST cohort. [file crc-23-0247-s09.pdf]

**SUPPLEMENTAL FIG 8:** Social and environmental factors

A

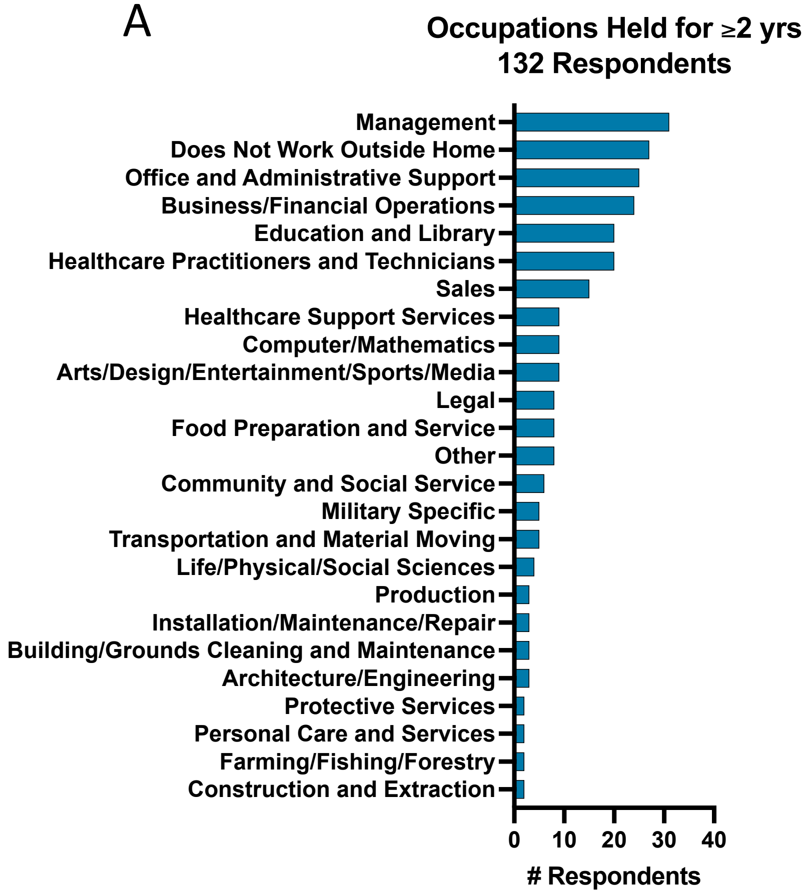


B

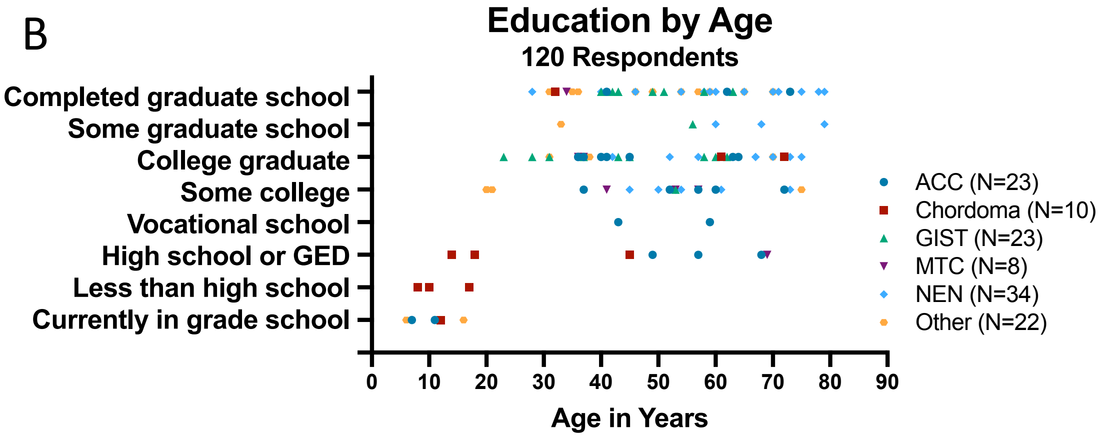


C

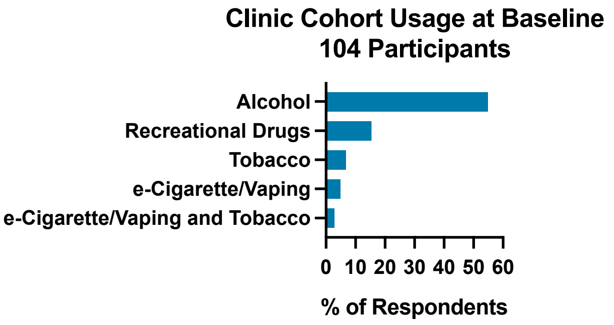


D

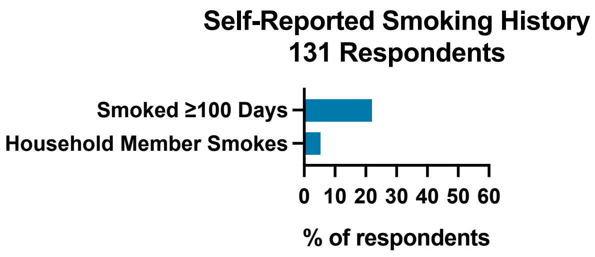

Supplemental Figure 8: Example of social and environmental characteristics of the NHRST cohort. (A) Occupations held outside of the home for at least two years in 132 responding participants. (B) Highest level of education attained (y-axis) in 120 responding participants by age (x-axis) across the tumor types. (C) Alcohol, recreational drug use, and smoking history of the clinic cohort collected during physical exam medical history interviews at NIHCC. (D) Self-reported data on tobacco exposure collected from 131 respondents at enrollment.
