# Supplementary material for: Longitudinal Natural History Study of Children and Adults with Rare Solid Tumors: Initial Results for First 200 Participants
Source: Cancer Res Commun. 2023 Dec 6;3(12):2468–82. doi: 10.1158/2767-9764.CRC-23-0247 (PMC10699159; doi:10.1158/2767-9764.CRC-23-0247)
Supplement: Supplementary Fig 9 — Other health issues reported in 3 or more participants out of 115 respondents by tumor type. [file crc-23-0247-s10.pdf]

SUPPLEMENTAL FIG 9: Self-reported health issues

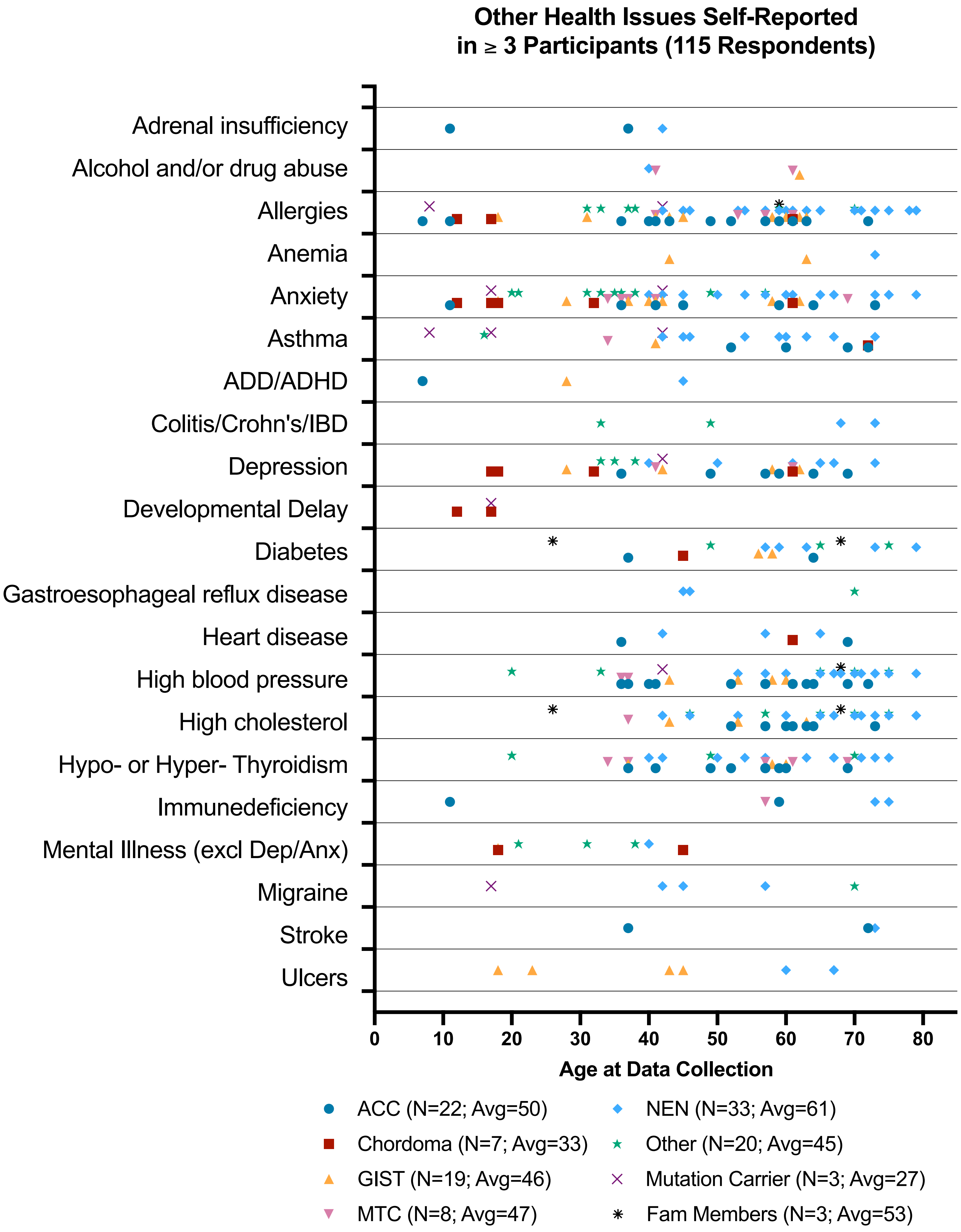

Supplemental Figure 9: Other health issues reported in 3 or more participants out of 115 respondents by tumor type. The x-axis shows respondents age in years at the time of data collection. The y-axis lists the various health problems reported. The average age for each tumor group is given in the legend.
