# Supplementary material for: Longitudinal Natural History Study of Children and Adults with Rare Solid Tumors: Initial Results for First 200 Participants
Source: Cancer Res Commun. 2023 Dec 6;3(12):2468–82. doi: 10.1158/2767-9764.CRC-23-0247 (PMC10699159; doi:10.1158/2767-9764.CRC-23-0247)
Supplement: Supplementary Fig 10 — Tumor-directed treatments. [file crc-23-0247-s11.pdf]

SUPPLEMENTAL FIG 10: Tumor treatments received at initial diagnosis and prior to enrollment

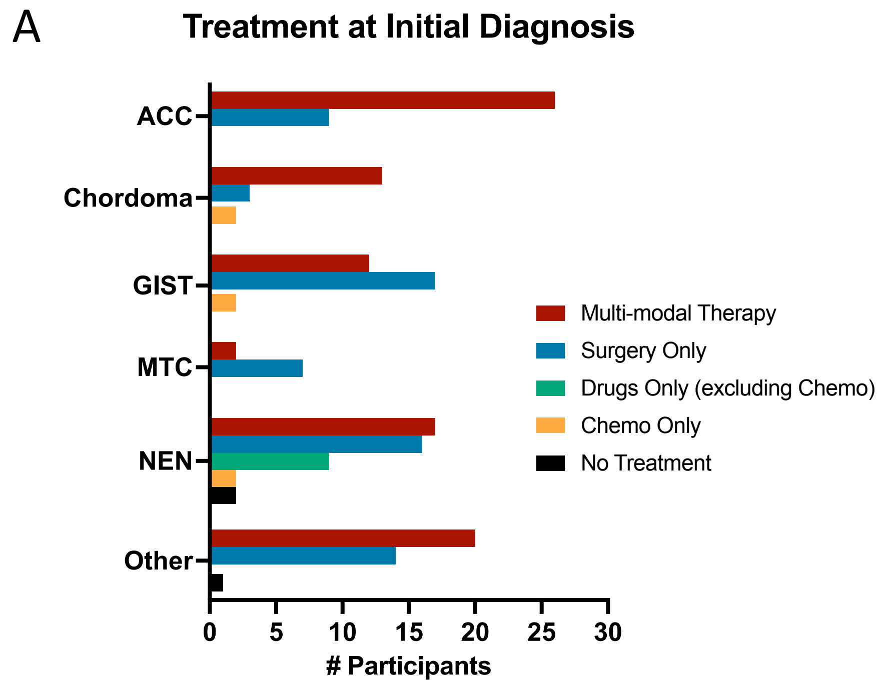

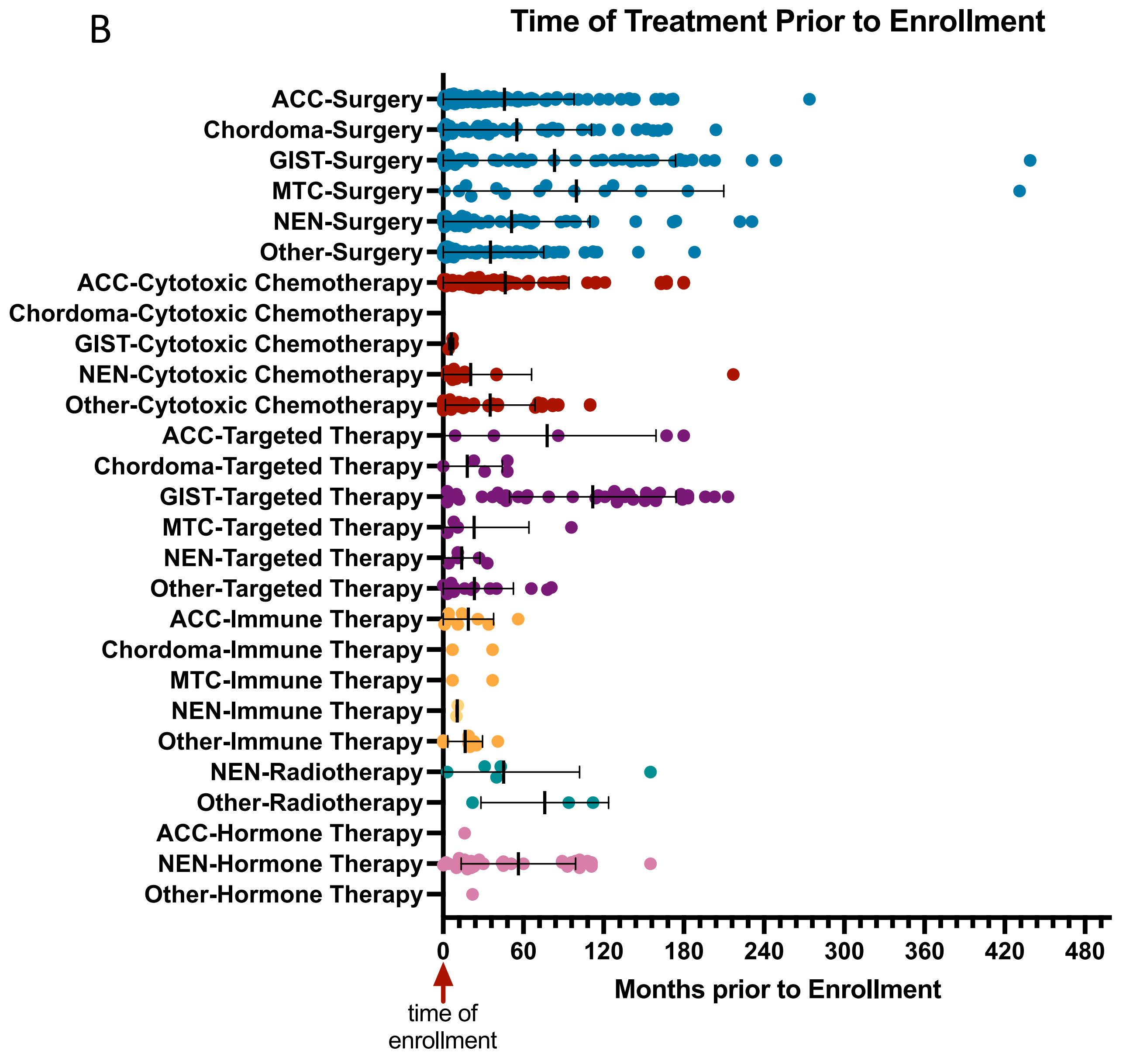

Supplemental Figure 10: Tumor-directed treatments. (A) Treatment at initial diagnosis comparing multi-modal therapy (combination of more than one category of treatment), surgery only, medical treatments excluding chemotherapy only, chemotherapy only, or no treatment. Treatments classified according to BC Cancer Pharmacology Education Program. (B) Tumor treatments received by patients prior to enrollment. Surgery was the most common treatment across all tumors and many participants began receiving treatment years before enrollment. Lines and error bares indicate mean and standard deviation. Medical records were extracted for 177 participants with tumors (ACC N=35, chordoma N=19, GIST N=32, MTC N=9, NEN N=47, Other N=35).
