# Supplementary material for: Longitudinal Natural History Study of Children and Adults with Rare Solid Tumors: Initial Results for First 200 Participants
Source: Cancer Res Commun. 2023 Dec 6;3(12):2468–82. doi: 10.1158/2767-9764.CRC-23-0247 (PMC10699159; doi:10.1158/2767-9764.CRC-23-0247)
Supplement: Supplementary Fig 11 — Anxiety and depression over time. [file crc-23-0247-s12.pdf]

SUPPLEMENTAL FIG 11: Anxiety and depression from time of diagn**osis**

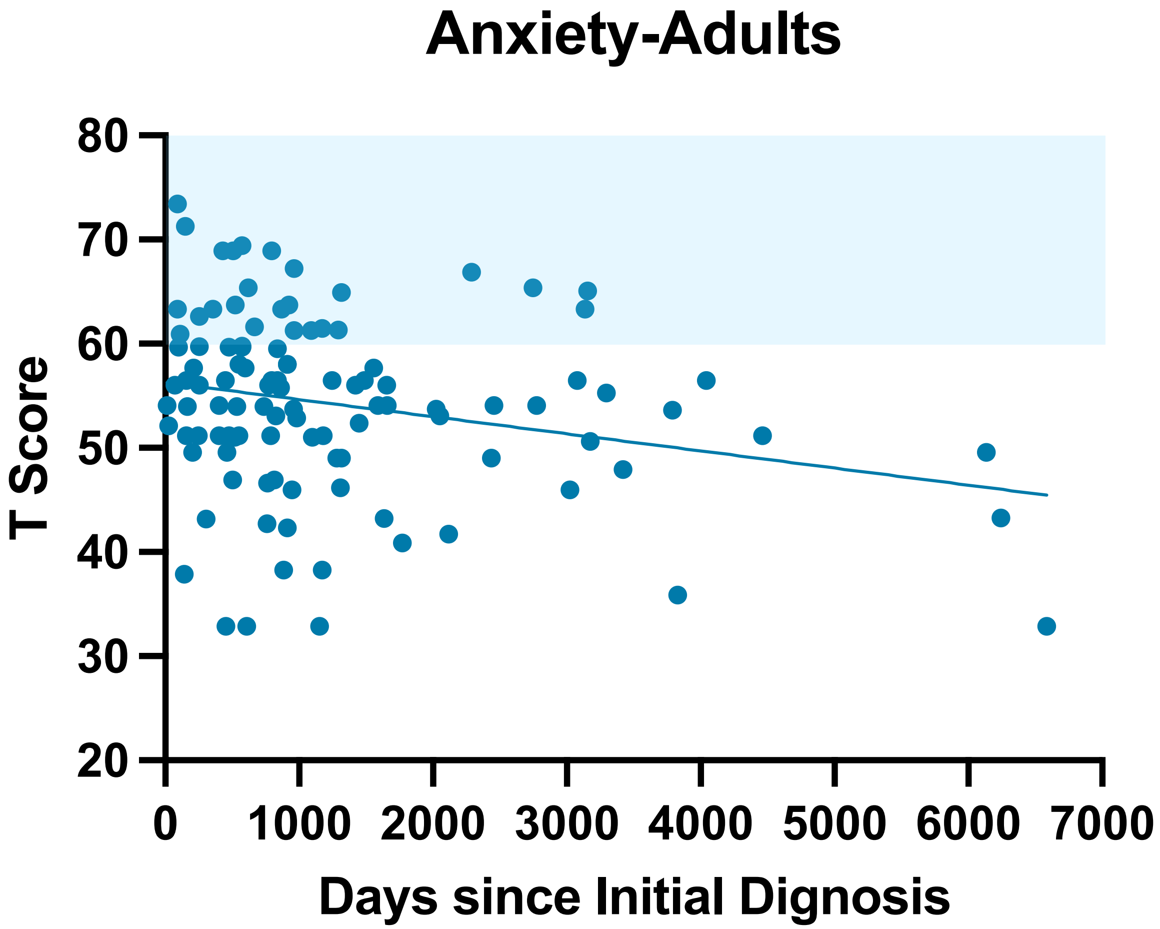

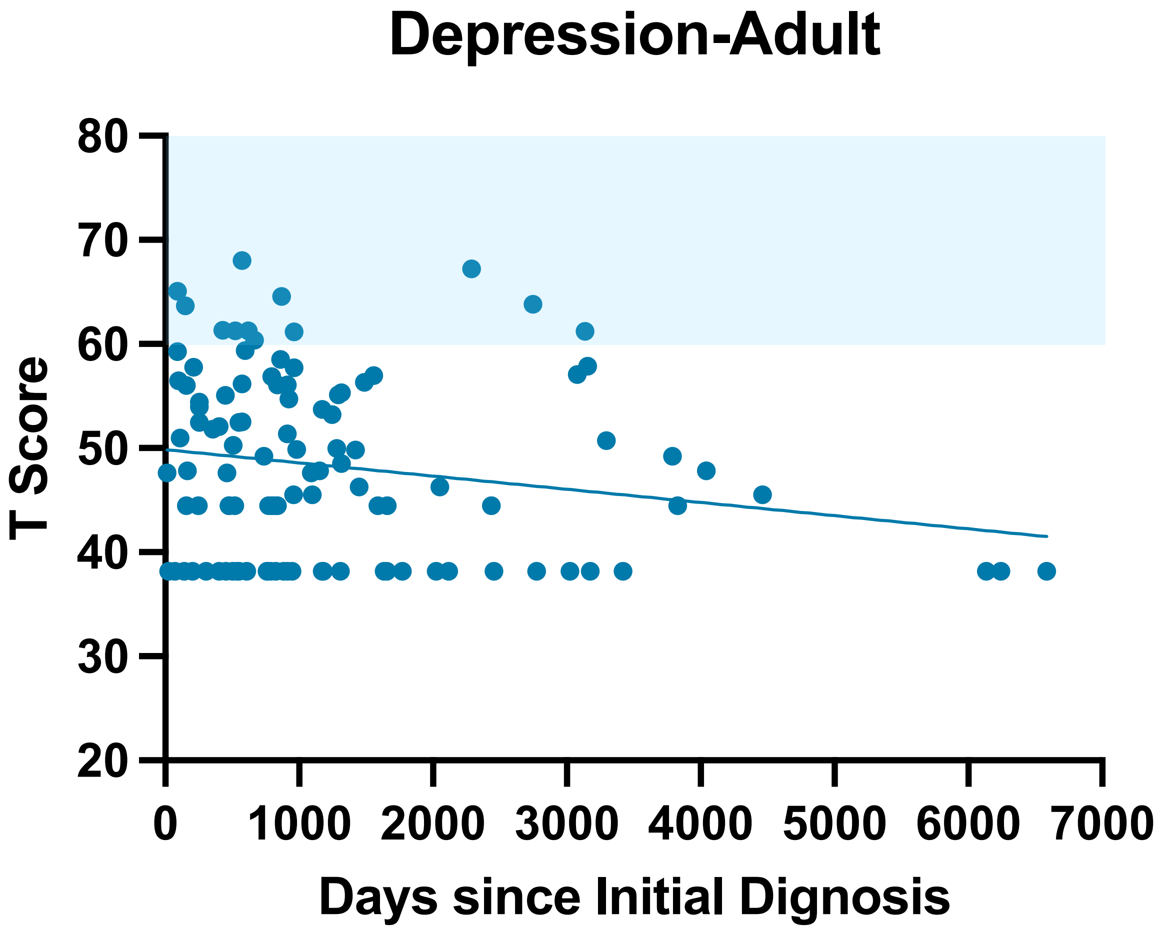

Supplemental Figure 11: Anxiety and depression over time. The T score for anxiety (A) or depression (B) reported by participants with rare tumors (y-axis) is graphed against the days since the participants initial diagnosis (i.e. how long they have lived with a tumor diagnosis) (x-axis). CS levels of anxiety and depression are indicated by the blue box. Anxiety and depression rates trend lower as participants are further from the time of initial diagnosis.
