# Supplementary material for: Longitudinal Natural History Study of Children and Adults with Rare Solid Tumors: Initial Results for First 200 Participants
Source: Cancer Res Commun. 2023 Dec 6;3(12):2468–82. doi: 10.1158/2767-9764.CRC-23-0247 (PMC10699159; doi:10.1158/2767-9764.CRC-23-0247)
Supplement: Supplementary Fig 12 — Success of TSO500 panel sequencing depending on age of formalin-fixed paraffin-embedded (FFPE) tissue. [file crc-23-0247-s13.pdf]

SUPPLEMENTAL FIG 12: Effect of FFPE sample age of success of gene panel sequencing

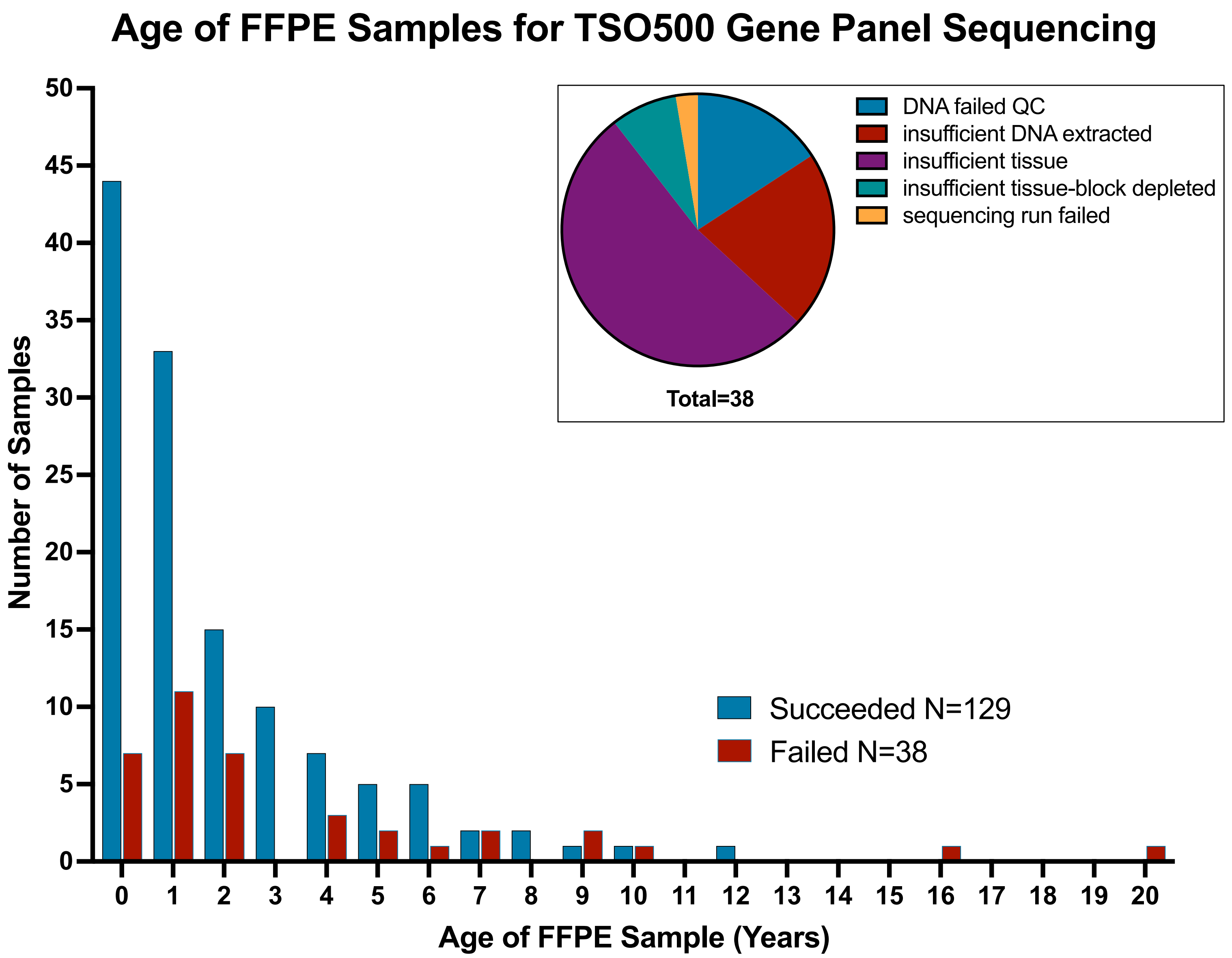

Supplemental Figure 12: Success of TSO500 panel sequencing depending on age of formalin-fixed paraffin-embedded (FFPE) tissue. The number of samples submitted that returned results (blue) and the number of samples that failed sequencing (red) are plotted against the age of the sample in years. The inset graph shows the distribution of reasons for assay failure.
